# Premating barriers in young sympatric snail species

**DOI:** 10.1038/s41598-021-84407-2

**Published:** 2021-03-11

**Authors:** Arina L. Maltseva, Marina A. Varfolomeeva, Arseniy A. Lobov, Polina O. Tikanova, Egor A. Repkin, Irina Y. Babkina, Marina Panova, Natalia A. Mikhailova, Andrei I. Granovitch

**Affiliations:** 1 Department of Invertebrate Zoology, St Petersburg State University, St Petersburg, Russia; 2 Laboratory of Regenerative Biomedicine, Institute of Cytology Russian Academy of Sciences, St Petersburg, Russia; 3 Institute of Molecular Biotechnology of the Austrian Academy of Sciences (IMBA), Vienna, Austria; 4 Department of Marine Sciences - Tjärnö, University of Gothenburg, Gothenburg, Sweden; 5 Centre of Cell Technologies, Institute of Cytology Russian Academy of Sciences, St Petersburg, Russia

**Keywords:** Behavioural ecology, Evolutionary ecology

## Abstract

Sympatric coexistence of recently diverged species raises the question of barriers restricting the gene flow between them. Reproductive isolation may be implemented at several levels, and the weakening of some, *e.g.* premating, barriers may require the strengthening of the others, *e.g.* postcopulatory ones. We analysed mating patterns and shell size of mates in recently diverged closely related species of the subgenus *Littorina Neritrema* (Littorinidae, Caenogastropoda) in order to assess the role of premating reproductive barriers between them. We compared mating frequencies observed in the wild with those expected based on relative densities using partial canonical correspondence analysis. We introduced the fidelity index (FI) to estimate the relative accuracy of mating with conspecific females and precopulatory isolation index (I_PC_) to characterize the strength of premating barriers. The species under study, with the exception of *L. arcana*, clearly demonstrated preferential mating with conspecifics*.* According to FI and I_PC_, *L. fabalis* and *L. compressa* appeared reliably isolated from their closest relatives within *Neritrema*. Individuals of these two species tend to be smaller than those of the others, highlighting the importance of shell size changes in gastropod species divergence. *L. arcana* males were often found in pairs with *L. saxatilis* females, and no interspecific size differences were revealed in this sibling species pair. We discuss the lack of discriminative mate choice in the sympatric populations of *L. arcana* and *L. saxatilis,* and possible additional mechanisms restricting gene flow between them.

## Introduction

Reproductive isolation is a keystone of the biological concept of the species^1^. The emergence of at least partial reproductive barriers is generally accepted as a crucial event in the evolutionary history of species, allowing them not only to diverge but also to persist in time^2,3^. Depending on the stage of the reproduction process, reproductive barriers in internal fertilizers are classified into premating isolation (PMI; anything that affects the existing mating patterns), postmating prezygotic (PMPZ) isolation (mechanisms acting between copulation and zygote formation), and postzygotic isolation (aspects associated with the ability to develop, viability and fertility of hybrids)^2,4^.

Observations and theoretical models support the idea that PMI evolves faster than PMPZ and postzygotic mechanisms^5,6^. In the course of speciation, PMI originates from the phenomenon of assortative mating. Several proximate mechanisms contribute to assortative mating and PMI: ecological isolation, including habitat-preference and habitat-associated fitness isolation; temporal isolation, with a shift in the timing of reproduction; and sexual isolation, when mate choice affects mating pattern^1,2,7,8^. PMI through assortative mating is usually considered as a possible driving force of incipient species divergence in sympatry, affecting the likelihood of speciation events^9–14^. PMPZ reproductive isolation in animals is less studied than premating or postzygotic isolation^6^. However, a few studies on model systems show that a weakening of PMI may require the strengthening of PMPZ barriers, thus closing the gap in reproductive barriers^6,15^ (but see^16^).

In this study, we evaluate the total efficacy of PMI in a group of closely related sympatric species of intertidal gastropods of the genus *Littorina*. A high microhabitat diversity in the intertidal makes it possible for morphologically and ecologically diverse organisms to coexist and to engage in complex interactions^17–20^. Moreover, the environmental heterogeneity of the intertidal zone may be a driving force of ecological speciation^21,22^. Indeed, many of the species sympatrically inhabiting intertidal areas are phylogenetically close and must have formed as a result of recent ecological divergence events, *e.g. Fucus* algae^23–25^; *Urosalpinx* gastropods^26^; *Idotea* crustaceans^27^. Effective reproductive barriers may be expected in sympatric sister-species, because they have to be strongly isolated to persist either after sympatric speciation or in secondary contact after allopatry^2^. The primary role should belong to mechanisms of premating (or, in case of external fertilizers, prezygotic) isolation since they are the fastest-evolving ones^6^.

Species of the *Littorina,* the subgenus *Neritrema* (Littorinidae, Caenogasropoda), are a textbook example of recently diverged species occurring in sympatry. Five of them live together at the shores of European Northern Atlantic seas: *L. obtusata* (Linnaeus, 1758) and *L. fabalis* (W. Turton, 1825) (“obtusata” group of species) and *L. saxatilis* (Olivi 1792), *L. compressa* Jeffreys, 1865 and *L. arcana* Hannaford Ellis, 1978 (“saxatilis” group of species). All *Neritrema* species are dioecious polygamic internal fertilizers showing no obvious sexual dimorphism in shell shape^28^; males are the choosing sex, while females (or other partners, see below) are mostly passive during copulation^29–32^.

High-density populations of the *Neritrema* species sympatrically coexist in the intertidal zone. Being very close phylogenetically, they are characterized by similar biological and morphological features and are often regarded as cryptic species^33–36^. This raises a number of intriguing questions. How do these species maintain species identity? What ensures effective reproductive isolation between these evolutionarily young species? What is the relative importance of premating barriers in their reproductive isolation?

The mating patterns of Littorinids have been extensively studied, with the role of assortative mating as a possible driver of speciation being given special attention^30–32,37–49^. For example, research on *L. saxatilis* and *L. fabalis* has focused on the role of sexual selection in the divergence of ecotypes and the restriction of the gene flow between them^31,42,43,46,50^. However, to the best of our knowledge, no analysis of interspecific mating patterns involving all the five North Atlantic sympatric periwinkles in wild populations has ever been made.

In this study, we estimated the contribution of premating barriers to reproductive isolation between recently diverged species in nature by tracing interspecific copulatory activity among already diverged closely related species instead of examining assortative mating in populations at the initial stages of their divergence (*e.g*. between ecotypes). Such an approach makes it possible (a) to deal with true species after the speciation event rather than with specialized forms, such as eco- or morphotypes, which might never become species; (b) to consider several species at the same time, not only the most contrasting forms; (c) to assess an actual mating activity in a natural population rather than in laboratory experiments, the results of which are not always easy to extrapolate to natural conditions.

The objective of the study was to describe in detail the mating activity in the chosen model species in order to estimate the reliability of PMI and to assess the contribution of possible proximate mechanisms of PMI to reproductive isolation. To achieve this, we registered mating activity in natural populations of five sympatric *Neritrema* species in the Barents Sea taking into account the population characteristics such as species densities, distribution pattern, and sex ratio, and the individual characteristics such as shell size, maturity, and trematode infection. The precopulatory isolation index (I_PC_) based on the joint isolation index^51^ and I_PSI_^52^ was used to describe total PMI, while a newly suggested male fidelity index (FI) was used to show an excessive mating with conspecific females compared to the null hypothesis of random mating.

We aimed (a) to quantify the frequency of interspecific mating, first of all, whether males mate with heterospecific females; (b) to list all distinct passive partner types that can mate with males of the *Neritrema* species in the wild, and to compare the similarity of such mating spectra among species of active partners and among types of passive partners; (c) to characterize the accuracy of mating with conspecific females using the newly suggested male fidelity index (FI, prevalence of matings with conspecific females among all mating committed by males of a given species); (d) to assess deviations from a random mating pattern simultaneously for males of all the species and all the types of passive partners using partial canonical correspondence analysis^53,54^ (pCCA), which would allow one to highlight general patterns by partialling out the effects of background factors (*e.g*. site and intertidal level) on mating frequencies; (e) to quantify total PMI using the precopulatory isolation index I_PC_; (f) to evaluate sizes of copulating partners of different species to examine the potential importance of shell size in interspecies PMI.

## Materials and methods

### Collection of copulating pairs

Individuals of five *Littorina* species: *L. arcana*, *L. compressa, L. saxatilis* (“saxatilis” group) and *L. fabalis*, *L. obtusata* (“obtusata” group) involved in copulation were collected at two distant intertidal sites at the Barents Sea coast: Dalnye Zelentsy, Oscar Bay, Russia (69°07′01.0″ N 36°04′07.5″ E; 05–15 July 2015) and Kiberg, Varangerfjord, Norway (70°16′55.1″ N 30°58′27.6″ E; 16–25 June 2016 and 16–25 June 2017). Both sites were represented by the flat stony-gravel littorals, overgrown with macrophytes of similar species composition. The samples were taken during the spring–summer peak of reproductive activity of all the species involved in the study^33,55–57^.

Pairs were collected from a restricted area of the intertidal zone (~ 25 m wide), separately from the upper and lower parts, because these are inhabited by different sets of species. These parts were recognized based on biocenotic characteristics: the lower part is overgrown with fucoid macroalgae (including *Fucus vesiculosus*, *Ascophyllum nodosum* and *F. serratus*), while the upper part has large boulders and rocky ledges without macrophytes, smaller boulders and pebbles with some scattered clumps of *F. vesiculosus* (more details in Supplement S1). The two sampling parts within the intertidal zones did not contact, being 3–5 m apart.

**Figure 1:**
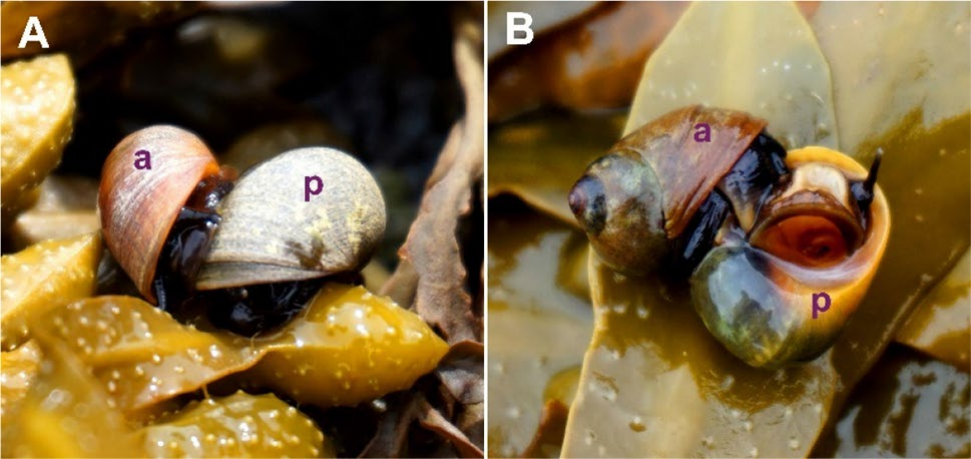
*Littorina obtusata* pair in copulation. (**A**) Typical position of copulating snails on the substrate. (**B**) A turned pair with an inserted penis visible. a—active partner (inserting penis); p—passive partner (accepting penis).

The snails were collected at low tide over several days. Copulating pairs were recognized based on their typical position (Fig. 1A). The event of penis insertion into the mantle cavity of a passive partner was verified visually while separating partners from each other (Fig. 1B, Supplement S2). All such events were considered as copulations though the penis insertion into the female bursa copulatrix (true copulation) could not be verified in this way. This means that true copulations were analysed together with copulation attempts. All the pairs of snails involved in the analysis are hereafter referred to as “copulating pairs”, following the conventional use of this term in similar studies on *Littorina* mating activity^30–32,37–44,46^. The pairs of snails considered as copulating were put into plastic bags, each pair into a separate bag, without marking either active (inserting penis) or passive (accepting penis) partners (Fig. 1B). Thus, it was impossible to determine later which of the partners was the active one in male-male pairs. The collected pairs were transferred to the laboratory, where their shell height was measured with calipers. After that, the snails were dissected to determine their maturity (based on well-developed genitalia and auxiliary glands), sex, trematode infection (based on the presence of parthenitae stages in the tissues of hepatopancreas), and the species.

In total, we collected 317 copulating pairs: 139 pairs at Dalnye Zelentsy in 2015 (91 at the upper and 48 at the lower intertidal), 89 pairs at Kiberg in 2016 (26 at the upper and 63 at the lower intertidal), and 89 pairs in Kiberg in 2017 (40 at the upper and 49 at the lower intertidal). The copulating pairs were categorised by the type of active and passive partner to obtain observed copulation frequencies. Male-male pairs, in which it was impossible to determine which of the partners was the active one, were recorded as 0.5 counts for both possible combinations.

Species identification of the *Neritrema* snails is notoriously difficult and requires considerable skills and an intimate knowledge of the molluscan morphology and anatomy. Morphological identification was performed by experienced specialists (Andrei Granovitch and Natalia Mikhailova) based on the diagnoses from the fundamental review^33^ and previous studies of these species^36,58–61^.

**Figure 2:**
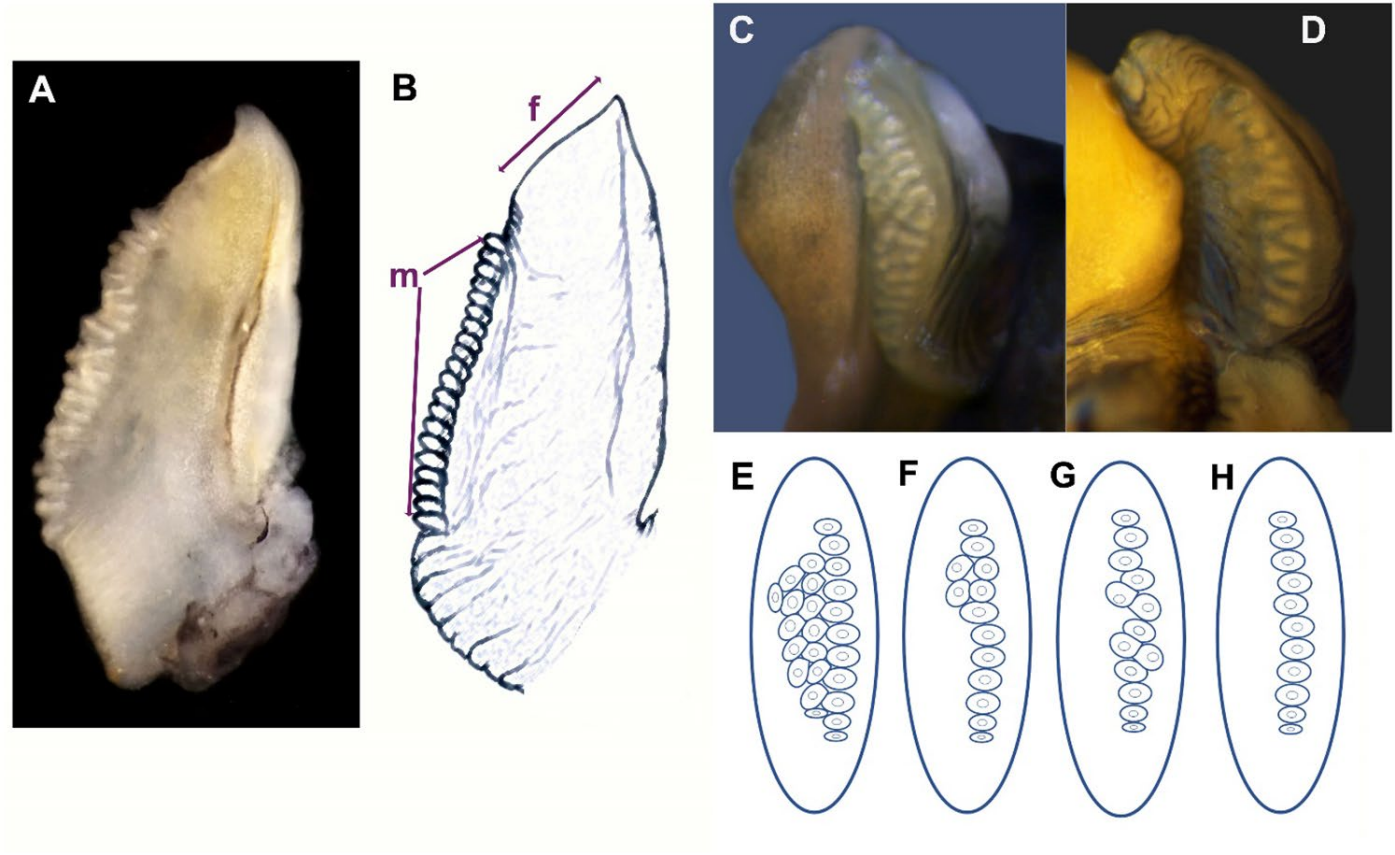
Morphology of a *Littorina* penis. (**A**) A photograph of *L. saxatilis* penis; (**B**) a scheme of a penis showing features important for identification—filament (f) and a row of mamilliform glands (m); (**C**–**H**) examples of penises with different numbers of gland rows; (**C**) multiple rows corresponds to scheme (**E**) (identified as *L. arcana)*; (**D**) 1.5 rows (there are no glands in an opposite position, although several glands are out of the row) corresponds to scheme (**G**) (identified as *L. saxatilis* 1.5, presumably hybrids between *L. arcana* and *L. saxatilis*); (**F**) two rows of penial glands, as two pairs of glands are in opposite position (identified as *L. arcana*); (**H**) one even row of penial glands (identified as *L. saxatilis* 1 row).

Three species of the “saxatilis” group (*L. compressa*, *L. arcana* and *L. saxatilis*) have a very similar morphology. Females can be distinguished by their reproductive system: females of *L. compressa* and *L. arcana* are oviparous, whereas *L. saxatilis* is ovoviviparous. They also differ in the structure of the distal parts of the female reproductive system, that is, the relative size of the albumen, capsule and jelly glands, as well as in the size and shape of the bursa copulatrix, which is broad and long in *L. arcana* and short and slim in *L. compressa*. Species identification of males of *L. saxatilis* and *L. arcana* was based on penis morphology^33,62^: the number of mamilliform glands and the shape of a filament (Fig. 2). Two or more rows of small numerous mamilliform penial glands and triangular filament were definitive for *L. arcana* (Fig. 2C,E,F); the distribution of glands on the penis surface was interpreted as two or more rows only if at least two glands were opposite to each other (Fig. 2F). Males with one row of large distal glands (not more than six in number) and a short filament were identified as *L. compressa*. Males with one row of numerous (more than six) small mamilliform penial glands and a triangular filament were classified as *L. saxatilis* 1 row (Fig. 2A,B,H). If one or more glands were out of the row, but not in an opposite position (in an intermediate position), such males were considered in a separate category, *L. saxatilis* 1.5 row (Fig. 2D,G), as possible hybrids between *L. saxatilis* and *L. arcana*^59^.

At present, there is no reliable specific-specific marker for identification of males of *L. saxatilis* and *L. arcana* by genotyping. Besides, some of the females with *L. arcana* morphology could have been hybrids^63^. However, the frequency of hybrids between *L. saxatilis* and *L. arcana* in the natural populations is probably very low. It was estimated to be < 2% by Warwick et al.^63^, while Stankowski et al.^64^ found no hybrids at all among 3,092 snails analysed.

Two species of the “obtusata” group (*L. obtusata* and *L. fabalis*) are oviparous and have a similar shell morphology in the study sites. The females of these species were identified based on the differences in the morphology of the bursa copulatrix, which is long, reaching up the albumen gland in *L. obtusata* and short and almost indiscernible in *L. fabalis*. The males of these two species differ in the number and shape of mamilliform glands and the morphology of the penial filament. The penis of *L. fabalis* has not more than six relatively large glands, located in the middle part of the organ; the filament is thin and long and may be longer than the basal penis part. The males with several rows of small glands on the penis with a short and thick filament were identified as *L. obtusata*^33^.

Immature or trematode-castrated (ITC) individuals were impossible to identify to the species level due to absence of well-developed reproductive organs (often even sex-identifying organs are underdeveloped or resorbed). These individuals were attributed only to their species group (“saxatilis” or “obtusata”), based on shell features^33^. These two groups were considered together due to their unidentified species/sexual status.

### Description of *Littorina* species composition on the shore

Snails were sampled from randomly positioned square frames with an area of 0.04 m^2^ (20 cm × 20 cm) from the upper (six replicate samples) and the lower (ten replicate samples) parts of the intertidal zone at each study site. A greater number of replicate samples at the lower intertidal was associated with the fact that its area was approximately 1.5 times larger than that of the upper intertidal. All the snails within the quadrat were collected, put into a plastic bag and transported to the laboratory, where they were measured, identified down to the species and subjected to size measurements as well as to assessment of maturity, sex, and trematode infection as described above.

The species composition of the snails was different in the upper and the lower parts of the intertidal zone. All the five species inhabited the lower intertidal, but only *L. arcana*, *L. saxatilis* and *L. obtusata* were recorded in the upper intertidal (*L. obtusata* was absent there in the Dalnye Zelentsy, 2015) (Supplement S3). For this reason, the mating patterns of the snails from the upper and the lower intertidal zone were analysed separately. Mean relative densities were used to calculate expected copulation frequencies under the random mating hypothesis (see below).

### Statistical analysis

Our aim was to estimate typical frequencies of potentially productive and unproductive matings in sympatric populations of the *Littorina (Neritrema)* species. We defined potentially productive matings as matings with conspecific females and unproductive mating as matings with any other partners, with a caveat that matings with some heterospecific females could be productive. We assumed that a total effect of PMI could be due to a combination of several phenomena such as reproductive timing (differential mating activity of males), habitat-preference and adaptation, sexual selection and isolation, but not to differentially mating preferences or sexual selection per se, which require a different sampling design and use of other estimators^47,48,52,65,66^. Disentangling the effects of sexual selection and isolation can be very informative when applied to population studies, and their estimators have been developed (PSS and PSI^52^). When considering multiple species, however, sexual selection and isolation cannot be differentiated due to strong effects of *e.g*., habitat-preference, on observed mating pattern, the so-called “scale of choice” problem^47,65,66^. Therefore, in our study we focused on the total effects of all PMI mechanisms, including non-homogeneous spatial distribution, reproductive timing, and mechanisms of mate choice.

To test whether *Littorina* (*Neritrema*) males mate at random with any of the possible categories of passive partners, we modelled random mating (Table 1) where all the *Neritrema* snails available at a particular site and intertidal level were considered as potential passive partners, including males, females, or ITC individuals of all the five species. Since female periwinkles appear to play a passive role in mating^29–31^ and since there is no evidence that any potentially passive mates actively avoid copulation, we assumed that all individuals older than one year (identified by presence of the first annual growth ring on a shell) were potential partners (we have never observed younger individuals in copulation). Under the null hypothesis of random mating, the probability of being a passive partner in copulation is equal among snails at a particular site and intertidal level. Therefore, frequencies of potential passive partners corresponded to the ratio of respective categories in the populations, which were estimated from the same 0.04 m^2^ quadrats that were used to assess *Littorina* species composition.

**Table 1:** Calculation of expected frequencies in the mating model used in the study.

		Passive partners (expected frequencies from population frequencies)		
		A’	B’	C’
Active male partners (expected frequencies from copulating pairs)		*P*_*A*′_ = *A′*/(*A′* + *B′* + *C′*)	*P*_*B*′_ = *B′*/(*A′* + *B′* + *C′*)	*P*_*C*′_ = *B′*/(*A′* + *B′* + *C′*)
a	*P*_*a*_ = *a*/(*a* + *b*)	*S*_*aA′*_ = *P*_*a*_* P*_*A*′_* t*	*S*_*aB′*_ = *P*_*a*_* P*_*B*′_* t*	*S*_*aC′*_ = *P*_*a*_* P*_*C*′_* t*
b	*P*_*b*_ = *b*/(*a* + *b*)	*S*_*bA′*_ = *P*_*b*_* P*_*A*′_* t*	*S*_*bB′*_ = *P*_*b*_* P*_*B*′_* t*	*S*_*bC′*_ = *P*_*b*_* P*_*C*′_* t*

The process is illustrated with two types of active three types of passive partners; the same logic applies to a different number of partner types. *A*′, *B*′, *C*′—the number of passive partners of the types studied, counted from the population frequencies. *P*_*A*′_, *P*_*B*′_, *P*_*C*′_—probability to be a passive partner in copulation, estimated from the numbers in the population. a, b—numbers of active male partners of the types studied, counted from copulating pairs. *P*_*a*_ *P*_*b*_—probability of copulation for a given type of active male partner, estimated form the frequency of this type of partner in copulating pairs. *t = aA′ + aB′ + aC′ + bA′ + bB′ + bC′—tot*al observed number of copulating pairs. *S*_*aA*′_, *S*_*bA*′_, *S*_*aB*′_, *S*_*bB*′_ , *S*_*aC*′_, *S*_*bC*′_—the expected number of pairs in each combination of partners.

In periwinkles, males are the active partners making copulation attempts^29–32^. Obviously, males of different species may differ in the degree of copulative activity for various reasons (*e.g.* not all mature males may be ready for copulation at a given time and their fraction in each species may vary). This is why we calculated frequencies of active partners based on their ratio in detected copulating pairs. Finally, the expected number of copulating pairs was calculated as the product of frequencies of potential passive partners, frequencies of active partners, and the total number of observed mating pairs (Table 1). This design does not correspond to any published estimators.

Our model of random mating does not take into account any possible habitat preference/adaptation barriers or spatio-temporal activity patterns (while the differences in microbiotope preferences between species do exist^28^). Nevertheless, it estimates a hypothetical chance of males meeting partners of different species/status.

### Possible combinations of partner types in copulating pairs

We listed all the possible passive partner types that can mate with males of each of the species of the subgenus *Neritrema*, as well as all possible active partner types that can mate with different types of passive partners. A combination of partner types was recorded as present if it appeared with non-zero observed frequency among all registered mating pairs. The information on presence/absence of observed combinations of partner types was arranged in a table, with types of active partners in columns and types of passive partners in rows (see the subsection (1) General description of copulatory activity in the Results section). Pairwise differences in the lists of observed combinations of partner types were evaluated separately for types of active mates (columns) and types of passive mates (rows) using simple mismatch coefficient^67^ (SMC). SMC is a distance measure that corresponds to the simple matching coefficient, which is the conventional similarity measure for binary data^68^. SMC between the lists of possible passive partner types of active partners of types *i* and *j* is calculated as ${SMC}_{\left(i,j\right)}=\frac{{f}_{10}+{f}_{01}}{{f}_{11}+{f}_{10}+{f}_{01}+{f}_{00}}$, where *f*_10_ is the number of distinct passive partner types that were observed mating with active males of type *i*, but not with *j*; *f*_01_—were observed mating with active males of type *j*, but not with *i*; *f*_00_—were not observed mating with either types of active partners, *f*_11_—were observed mating with the both types of active partners. SMC between the lists of possible active partner types of passive partners was calculated in the same way. The two resulting symmetric distance matrices were clustered using Unweighted Pair Group Method with Arithmetic Mean (UPGMA) and plotted using dendextend package^69^ in R^70,71^.

Fidelity Index (FI) was proposed to measure the prevalence of heterosexual mating with conspecific partners (potentially productive matings) over mating with other types of partners (potentially unproductive ones) compared to the null expectation of random mating. It was calculated as a difference between the observed and the expected numbers of copulations with conspecific partners, divided by the sum of expected numbers of all copulations. For example, following the notation defined in Table 1, ${FI}_{aA{^\prime}}=\frac{aA^{\prime}-SaA{^\prime}}{{S}_{aA{^\prime}}+{S}_{aB{^\prime}}+{S}_{aC{^\prime}}}$ is the fidelity of males of type *a* to conspecific females *A’* (the difference of observed and expected frequencies of mating of males of type *a* with conspecific females *A’* is divided by the sum of expected frequencies of copulation of males of type *a* with conspecific and all heterospecific females). The possible values of fidelity index range from − 1 to 1 (-1 = avoidance, 0 = random mating, 1 = assortative mating). The index was calculated for males and females of each species within each year/site × level combination. Bootstrap with 10 000 iterations was used to calculate mean bootstrap values, standard deviations, and the two-tail probability of rejecting the null hypothesis of random mating (H_0_: FI = 0).

### Ordination in the space of sexual partners

Patterns of copulation in the species studied were described using partial Canonical Correspondence Analysis^53,54,72^ (pCCA) using vegan package^73^. pCCA is a χ^2^-based multivariate approach. This method has a certain advantage over traditional univariate methods of mating pattern analysis, because it allows one to visualise and compare multiple categories of partners simultaneously as a whole system. Thus, pCCA allows one to avoid multiple comparisons of mating frequencies. In addition, pCCa permits evaluation of possible deviation of observed frequencies from the frequencies expected under the null hypothesis, after removing the effect of background factors such as site and intertidal level, thus highlighting common and general patterns.

The observed copulation frequencies were arranged in a table with passive partners in rows and active partners in columns. Expected copulation frequencies for pCCA were calculated as described above, based on species- and sex-composition, and frequency of copulating males. In the pCCA procedure, the effect of the type of passive partner (combination of species and sex) was tested after removing the variation explained by the covariate (combination of site and year). Due to different species composition two separate analyses were performed on the data from the upper (157 pairs) and lower intertidal levels (149 pairs). Several categories of passive copulants (males of *L. arcana* and *L. saxatilis* with 1.5 rows of penial glands, and immature “saxatilis” snails) were excluded from the analysis of the data from the lower level in Kiberg in 2016 because these categories were absent in the field samples and so their expected mating frequencies could not be calculated. The effect of passive partner type on copulation frequencies was tested using Monte Carlo procedure with 10 000 permutations, restricted within strata defined by sampling site and year. The percentage of variance explained by each axis was obtained by dividing the canonical eigenvalues by the total inertia.

Degree of disassortative mating between pairs of *Littorina* species was assessed at each year/site and intertidal level using the precopulatory isolation index I_PC_. I_PC_ was computed as a joint isolation index^51,74^, except that the ratios of observed to expected frequencies of specific copulation types were used instead of raw counts. The ratios were also used when calculating I_PSI_, a metric proposed by Rolán-Alvarez & Caballero^52^. Previous research has shown that basing calculations on the ratios rather than the raw counts improves the statistical properties of isolation metrics^52,75^. Our metric (I_PC_) differs from I_PSI_, however, in that it estimates the combined effect of precopulatory isolation (*i.e.* sexual isolation and sexual selection) because the expected frequencies are calculated using both the composition and pairing frequencies, obtained from the null model of copulation described above, within each population. The value of I_PC_ varies between -1 and 1, where -1 indicates disassortative mating, 0 = random mating, and 1 = assortative mating. Only copulations with mature females were used for the analysis. *L. saxatilis* males with 1.5 rows of penial glands were excluded. Mean bootstrap values, standard deviations, and the two-tail probability of rejecting the null hypothesis of random mating (H_0_: I_PC_ = 0) were calculated using bootstrap with 10 000 iterations.

### The effect of shell size on mating patterns

Shell size of mating and non-mating individuals was summarized on boxplots to examine distribution of sizes. Kernel density plots were used to visualize overlap of the size-distributions of mating and non-mating snails.

Mean differences of partner shell sizes in heterosexual pairs (with con- or heterospecific females, and ITC individuals) were analysed to reveal any tendencies to mate with partners of a certain size. Size differences between partners were tested using paired *t*-test separately for each active partner species, year/site, and intertidal level (where ≥ 5 pairs were available).

Interdependence of partner shell sizes in all heterosexual pairs of *L. saxatilis* from the upper level (where the sample size was sufficient) was tested using a full-factorial 3-way permutational Analysis of Covariance (ANCOVA) in lmPerm package^76^. Active partner size was a response, and passive partner size, pair type (same-species *vs* other), year/site, and their interactions were used as predictors. Type III tests^77^ were run with 100 000 permutations.

Correlation of partner shell sizes in pairs with mature conspecific females was computed for *L. saxatilis*, *L. obtusata* and *L. fabalis* to test a possible importance of size in mate choice. Calculations were performed separately for different year/sites and intertidal levels. A test with 10 000 permutations was used to assess significance. To find out whether correlation of partner sizes in pairs with mature conspecific females could be a consequence of random mating within microareas (*e.g.* sample squares), a Monte Carlo simulation was performed. Random pairs of males with mature conspecific females were simulated from each of the quadrats and correlation of their sizes was computed. This procedure was repeated 10 000 times. *P*-value (the proportion of correlation coefficients with an absolute value greater or equal to the observed value) in this test can be interpreted as the probability to get correlation this strong when snails mate at random within the sample squares. To assess spatial segregation by size (another possible cause of correlation of partner sizes in the pairs with mature conspecific females), a permutation test was performed for *L. saxatilis* and *L. obtusata*, for which enough data were available. Analysis was performed separately for different year/sites and intertidal levels, following the procedure adapted from Erlandsson and Johannesson^39^. Snails were divided into two size groups: small and large relative to local median shell height. Small snails were assigned rank 0 while large snails—rank 1. The variance of ranks was calculated for each sample, and the mean variance of ranks was the test statistic. Data on the number of small and large snails were permuted 10 000 times to compute the empirical distribution of average variance. Low observed mean variance would indicate spatial segregation by size. *P*-values were computed as a fraction of permutations that produced lower mean variance than the originally observed value. Plots were produced using ggplot2 package^78^.

## Results

### General description of copulatory activity

In total, 317 copulating pairs of five *Littorina* species were collected from two collection sites at the Barents Sea coast during three summer seasons. Most of the copulating snails were identified as *L. saxatilis* and *L. obtusata* (43% and 22% of all copulating snails; Supplement S5). This was not surprising because those species were the most abundant in the study sites (Supplement S3).

The proportion of unproductive copulations was different between the species groups. Among all the recorded copulations, 60% were productive (191 pairs with conspecific female) and 40% were unproductive (19 [6%] with heterospecific females; 59 [19%] with conspecific or heterospecific males; and 48 [15%] with ITC individuals (Table 2). Interestingly, males of the “saxatilis” group were involved in potentially unproductive mating far more often (109 of 206 pairs, 53%) than those of the “obtusata” group (24 of 111 pairs, 22%) (Table 2), though the sex ratio in both species-groups in all analysed populations was roughly the same (Supplement S3).

**Table 2:** Mating patterns of active male *Littorina* snails grouped by site, intertidal level and species.

Site	Level	Active male species	Total	Conspecific female	Heterospecific female	Immature or castrated	Male (any species)
D. Zelentsy, 2015	Upper	*L. saxatilis*	63.5	41 (64.6)		10 (15.7)	12.5 (19.7)
		*L. saxatilis* (1,5 row)	12.5	6 (48)		1 (8)	5.5 (44)
		*L. arcana*	15		7 (46.7)	2 (13.3)	6 (40)
	Lower	*L. saxatilis*	17	10 (58.8)	1 (5.9)	4 (23.5)	2 (11.8)
		*L. arcana*	2.5		2 (80)		0.5 (20)
		*L. compressa*	3	3 (100)			
		*L. obtusata*	23.5	19 (80.9)			4.5 (19.1)
		*L. fabalis*	2	1 (50)	1 (50)		
Kiberg, 2016	Upper	*L. saxatilis*	21	13 (61.9)		6 (28.6)	2 (9.5)
		*L. saxatilis* (1,5 row)	3.5	2 (57.1)		1 (28.6)	0.5 (14.3)
		*L. arcana*	1.5				1.5 (100)
	Lower	*L. saxatilis*	17.5	9 (51.4)		5 (28.6)	3.5 (20)
		*L. saxatilis* (1,5 row)	7	4 (57.1)		2 (28.6)	1 (14.3)
		*L. arcana*	4		2 (50)	1 (25)	1 (25)
		*L. compressa*	1.5	1 (66.7)			0.5 (33.3)
		*L. obtusata*	29	27 (93.1)			2 (6.9)
		*L. fabalis*	4	2 (50)	1 (25)	1 (25)	
Kiberg, 2017	Upper	*L. saxatilis*	22	8 (36.4)	2 (9.1)	8 (36.4)	4 (18.2)
		*L. saxatilis* (1,5 row)	3.5	1 (28.6)		2 (57.1)	0.5 (14.3)
		*L. arcana*	2	1 (50)	1 (50)		
		*L. obtusata*	12.5	7 (56)		1 (8)	4.5 (36)
	Lower	*L. saxatilis*	4	1 (25)	1 (25)		2 (50)
		*L. compressa*	5	4 (80)			1 (20)
		*L. obtusata*	22	17 (77.3)		1 (4.5)	4 (18.2)
		*L. fabalis*	18	14 (77.8)	1 (5.6)	3 (16.7)	

Total number of copulations of a particular active partner type, as well as the number (and percentage) of copulations grouped by passive partner category is given. Male-male copulations were registered as 0.5 for each of the partners, because it was impossible to determine which of them was active. The category *L. saxatilis* (1.5 row) includes male *L. saxatilis* with 1.5 rows of penial glands (presumably hybrids of *L. saxatilis* and *L. arcana*); both *L. saxatilis* and *L. arcana* females were considered their conspecifics. Empty table cells indicate that corresponding mating combinations were not detected.

Males and females were recognized by their partners with a different accuracy. Not all possible combinations were detected. For example, there were no registered pairs of any males of “saxatilis” group with *L. obtusata* females, and vice versa, no pairs of *L. obtusata* males with any of the “saxatilis” group females, implying the reliability of premating barriers. At the same time, heterospecific male-male copulations were detected in all possible combinations (*L. arcana* + *L. obtusata*, *L. compressa* + *L. obtusata*, *L. saxatilis* + *L. obtusata*, Table 3). These differences in accuracy of male- or female-recognition may be explained by different cues involved in setting off copulation attempts with males and females. This agrees with the observation that among 210 (66%) male–female pairs only 19 (9%) pairs were between heterospecifics, while among 59 (19%) male-male pairs, there were 14 (24%) heterospecific pairs. A possible interpretation is that a male recognizes the species of a passive partner more accurately when mating with a female. This is consistent with the generally closer ordination of males and ITC to the origin compared to females on CCA-plots (Supplement S8). However, it is applicable not to all the species studied as no male-male pairs, either con- or heterospecific, were registered in case of *L. fabalis*.

**Table 3:** Observed combinations of partner types in mating pairs.

Passive partner	Active partner					
	*L. saxatilis*	*L. saxatilis* 1.5 row	*L. arcana*	*L. compressa*	*L. obtusata*	*L. fabalis*
*L. saxatilis* female	+	+	+	−	−	+
*L. saxatilis* male	+	+	+	−	+	−
*L. saxatilis* male (1.5 row)	+	+	+	−	−	−
*L. arcana* female	+	−	+	−	−	−
*L. arcana* male	+	+	+	−	+	−
*L. compressa* female	+	−	−	+	−	−
*L. compressa* male	−	−	−	+	+	−
*L. obtusata* female	−	−	−	−	+	+
*L. obtusata* male	+	−	+	+	+	−
*L. fabalis* female	+	−	−	−	−	+
'saxatilis'	+	+	+	−	−	−
'obtusata'	−	−	−	−	+	+

The category *L. saxatilis* (1.5 row) includes male *L. saxatilis* with 1.5 rows of penial glands (presumably hybrids of *L. saxatilis* and *L. arcana*). The categories “obtusata” and “saxatilis” include immature or castrated individuals of the corresponding cryptic species group.

**Figure 3:**
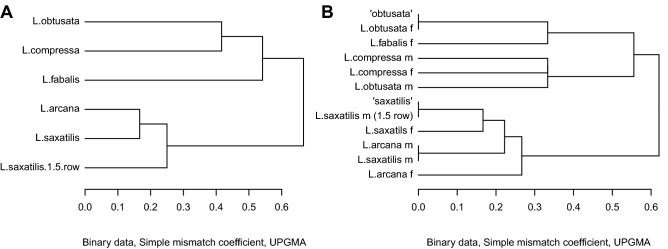
Clustering of observed combinations of partner types. Distances indicate dissimilarity of the lists of all observed partner types for active (**A**) and passive (**B**) mates (simple mismatch coefficient; Mirkin, 1996). The category *L. saxatilis* (1.5 row) includes *L. saxatilis* males with 1.5 rows of penial glands (presumably hybrids of *L. saxatilis* and *L. arcana*). The categories “obtusata” and “saxatilis” include immature or castrated individuals of the corresponding cryptic species group.

Mating patterns of *L. compressa* were different from its close relatives *L. saxatilis* and *L. arcana*. Individuals of *L. saxatilis* and *L. arcana* were encountered in similar mating combinations, both as active and passive partners, as evidenced by their close grouping on the dendrograms (Fig. 3, Table 3). *L. compressa* grouped with *L. obtusata* and *L. fabalis* (Fig. 3) and appeared reproductively isolated from the two other species of the “saxatilis” group: active males of *L. compressa* did not mate with either passive adults of *L. saxatilis* and *L. arcana* or with ITC “saxatilis” snails (all these categories co-occurred with *L. compressa* in the populations), while passive individuals of *L. compressa* mated only with conspecifics or with *L. saxatilis* out of all the species of the “saxatilis” group (Table 3). Mating combinations within the *L. compressa*, *L. fabalis*, *L. obtusata* cluster were more dissimilar than within the *L. saxatilis* and *L. arcana* cluster (Fig. 3), which also indicates a strong reproductive isolation within the former cluster.

### Prevalence of copulations with conspecific females

The FI was calculated for males of each species (as choosing partners, see above) to assess the degree of the mate choice accuracy (the prevalence of matings with conspecific females). The lower the FI value, the more often males of a particular species commence potentially unproductive copulation attempts. Similarly, this index was calculated for the females to find out how frequently they were engaged by conspecific males (Supplement S7). On average, the FI values of the “obtusata” group males exceeded those of *L. saxatilis* and especially *L. arcana* (Table 4, Supplement S7), whereas males of *L. compressa* demonstrated the highest fidelity among all the five species (Table 4) and the lowest rate of unproductive mating (16%; Table 2). However, these estimates should be treated with caution due to the comparatively low number of pairs analysed (11).

**Table 4:** Fidelity index (FI) of male *Littorina* snails grouped by species, intertidal level and year/site.

Species	Level	D. Zelentsy, 2015	Kiberg, 2016	Kiberg, 2017
*L. saxatilis*	Upper	0.48 ± 0.06, p < 0.001	0.51 ± 0.11, p < 0.001	0.27 ± 0.11, p = 0.005
	Lower	0.48 ± 0.13, p < 0.001	0.45 ± 0.13, p < 0.001	0.18 ± 0.22, p = 0.780
*L. arcana*	Upper	− 0.04 ± 0.02, p = 0.183	0.03 ± 0.16, p = 0.204	0.34 ± 0.34, p = 0.680
	Lower	0.03 ± 0.11, p = 0.182	− 0.01 ± 0.07, p = 0.173	
*L. compressa*	Lower	0.62 ± 0.27, p = 0.066	0.37 ± 0.35, p = 0.692	0.67 ± 0.22, p = 0.035
*L. obtusata*	Upper			0.48 ± 0.15, p = 0.002
	Lower	0.71 ± 0.09, p < 0.001	0.52 ± 0.08, p < 0.001	0.38 ± 0.11, p = 0.005
*L. fabalis*	Lower		0.38 ± 0.26, p = 0.306	0.73 ± 0.11, p < 0.001

FI measures prevalence of mating of males with conspecific females. It is computed as difference of observed and expected numbers of heterosexual conspecific copulations with males of a given species, divided by the total number of copulations with males of that species. Values of FI vary from − 1—avoidance, through 0—random mating, to 1—assortative mating. FI was not computed for *L. saxatilis* males with 1.5 rows of penial glands. Bootstrap means ± standard deviations are given; *p*—the two-tail probability of rejecting H_0_: FI = 0 in the test using bootstrap with 10 000 iterations.

### Ordination in the space of sexual partners

Partial canonical correspondence analysis (pCCA, Supplement S8) was applied to evaluate a possible deviation of observed mating frequencies from the ones expected under the assumption of random mating (calculated based on species- and sex-ratio in the natural populations). The pCCA ordination of passive and active partners generally confirmed the tendencies outlined above. Firstly, the mating frequencies of female passive partners differed more strongly from the random pattern than the mating patterns of the other categories. Secondly, *L. obtusata, L. fabalis* and *L. compressa* males were clearly attracted to conspecific females.

*Littorina obtusata* was reliably isolated from both the species of the “saxatilis” group and from *L. fabalis*. This was confirmed by pCCA ordination (Supplement S8) and by a high value of the isolation index within the “obtusata” group (0.88 and 0.99 in Kiberg in 2016 and 2017, respectively; Table 5). *L. obtusata* males showed preferential mating with conspecific females and with conspecific males, the latter being evident in the upper littoral zone.

**Table 5:** Precopulatory Isolation Index within and between *Littorina* “saxatilis” and “obtusata” species categorized by site and littoral zone.

Comparison	Species	Level	D. Zelentsy, 2015	Kiberg, 2016	Kiberg, 2017
Within “saxatilis” group	*L. saxatilis*—*L. arcana*	Upper	0.19 ± 0.21, p = 0.377	0.86 ± 0.34, p = 0.14	0.26 ± 0.52, p = 0.605
		Lower	0.03 ± 0.54, p = 0.879	− 0.14 ± 0.54, p = 0.541	
	*L. saxatilis*—*L. compressa*	Lower	0.89 ± 0.16, p = 0.003	0.91 ± 0.26, p = 0.066	0.94 ± 0.19, p = 0.012
	*L. arcana*—*L. compressa*	Lower	0.78 ± 0.55, p = 0.199	0.72 ± 0.6, p = 0.214	
Within “obtusata” group	*L. obtusata*—*L. fabalis*	Lower		0.88 ± 0.21, p = 0.023	0.99 ± 0.04, p < 0.001
Between groups	*L. obtusata*—*L. saxatilis*	Upper			0.98 ± 0.06, p < 0.001
		Lower	0.99 ± 0.03, p < 0.001	0.99 ± 0.04, p < 0.001	0.92 ± 0.23, p = 0.058
	*L. obtusata*—*L. arcana*	Upper			0.92 ± 0.23, p = 0.036
		Lower	0.86 ± 0.34, p = 0.112	0.9 ± 0.25, p = 0.015	
	*L. obtusata*—*L. compressa*	Lower	0.95 ± 0.14, p < 0.001	0.92 ± 0.24, p = 0.029	0.98 ± 0.09, p = 0.004
	*L. fabalis*—*L. Saxatilis*	Lower		0.95 ± 0.14, p = 0.004	0.58 ± 0.34, p = 0.096
	*L. fabalis*—*L. arcana*	Lower		0.83 ± 0.42, p = 0.128	
	*L. fabalis*—*L. compressa*	Lower		0.86 ± 0.35, p = 0.091	0.97 ± 0.07, p < 0.001

I_PC_ describes total premating isolation; its value varies between − 1 and 1 (− 1—disassortative mating, 0—random mating, 1—assortative mating). *L. saxatilis* males with 1.5 penial rows were excluded from the analysis. Bootstrap means ± standard deviations are given; *p*—the two-tail probability of rejecting H_0_: I_PC_ = 0 in the test using bootstrap with 10 000 iterations.

Males of *L. fabalis* apparently preferentially mated with conspecific females (Supplement S8), which corresponds to a high FI value of these males and a significant precopulatory isolation within the “obtusata” group (Table 5).

In the “saxatilis” group, males of *L. compressa* had a high FI value (Table 4) and were attracted to conspecifics according to pCCA results (Supplement S8). Although members of the “saxatilis” group in general showed low or non-significant I_PC_ values, on two occasions precopulatory isolation between *L. compressa* males and *L. saxatilis* was significant (Table 5).

**Figure 4:**
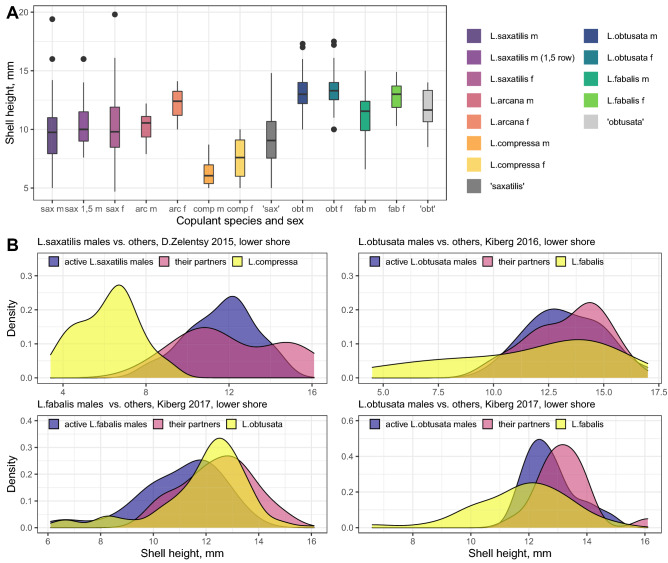
Shell height of copulating *Littorina* snails. Male-male pairs were excluded from the analysis. (**A**) All partners categorized by sex and species; data from different sites and levels were pooled. Dots indicate the values less or greater than 1.5 interquartile ranges from the median. (**B**) Shell height distributions illustrating potential for isolation by size in *L. saxatilis vs L. compressa*, *L. obtusata vs L. fabalis* (and vice versa); copulating males of the first species (violet), their passive partners (magenta, male-male pairs were excluded), and all collected individuals of the second species (yellow). All data on the size of mating and non-mating snails can be found in Supplement S9.

A peculiar mating pattern was found in the species pair *L. arcana* + *L. saxatilis*. Three categories of active partners were taken into account: *L. saxatilis* (1 row of penial glands), *L. arcana* (2 and more rows of penial glands) and 1.5 rows-males (presumably hybrids between *L. saxatilis* and *L. arcana*; for details see Methods section and^59^). Males of all the three categories exhibited a similar pattern of copulatory activity, being most often found in pairs with *L. saxatilis* females. This is why these three categories of males grouped very closely on pCCA biplots in both the upper and the lower intertidal (Fig. 4). While *L. saxatilis* males had moderately high FI values (Table 4), *L. arcana* males had negative FI values in four of the five cases, which implies more rare pairing with conspecific females than expected based on random encounters. Actually, out of the 13 pairs of *L. arcana* males with mature females, 12 were with *L. saxatilis* females and only one with *L. arcana* female. Similarly, males of *L. saxatilis* were mainly recorded in pairs with females of *L. saxatilis*, (82 of 86 pairs) and only rarely with females of the other species (2 pairs with *L. arcana*, 1 with *L. compressa*, 1 with *L. fabalis*; Table 2). The FI values for males with 1.5 penial rows could not be calculated because their species was uncertain. Nevertheless, all the 13 heterosexual copulations with mature individuals, registered for these males, involved *L. saxatilis* females only (Table 2). This unexpected “neglect” of *L. arcana* females by conspecifics as well as other species determined the low values of I_PC_ from the other species of the “saxatilis” group (Table 5).

### The effect of shell size on copulation

The size of copulating snails was compared to the size distribution in the field samples to test the randomness or assortative mating. The size may explain some of the copulatory patterns in the populations studied. Despite a considerable overlap of size ranges, both active and passive copulating snails of different species differed in their mean size (Fig. 4; Supplements S9–S14). For example, copulating individuals of *L. fabalis* and *L. obtusata* were on average larger than those of the species of the “saxatilis” group; copulating *L. obtusata* were usually slightly larger than *L. fabalis*; copulating *L. arcana* and *L. saxatilis* apparently exceeded *L. compressa* in size. The overlap in the latter case was rather low (Fig. 4B), suggesting that a mismatch in size might contribute to the reproductive isolation of at least *L. compressa* from *L. saxatilis* and *L. arcana*.

Males copulating with ITC individuals were on average smaller than those copulating with mature females (Supplement S11). This tendency was apparent for *L. saxatilis* and for *L. fabalis*. In heterosexual pairs of *L. fabalis* and *L. saxatilis* with 1.5 penial rows, the male was significantly smaller than its female mate (Supplement S14).

A significant correlation of partner sizes was observed for pairs involving *L. saxatilis* males, when the pairs with conspecific females and with other partners (heterospecific females or ITC individuals) were analysed together (Supplement S15). Some other tendencies related to roles of the shell size in the mating activity of the snails can be found in Supplements S9–S16.

## Discussion

In this study, we analysed natural mating activity in populations of five closely related periwinkle species *L. arcana*, *L. compressa*, *L. saxatilis*, *L. fabalis* and *L. obtusata*, living in sympatry in a large part of their European distribution. These species demonstrate a pronounced seasonality of reproduction in the Northern part of their distribution, with breeding seasons overlapping between the species^33,55–57^. We used the composition and frequencies of copulating pairs as a proxy for the description of the mating pattern and PMI strength. As expected for sympatric species, we observed that mating activity deviated significantly from the null hypothesis of random mating, which implies that interspecies PMI does function. However, the species varied in the efficacy of interspecies PMI. Below we hypothesize about the possible mechanisms underlying these barriers and the variation of their strength. Interestingly, the females were recognized more accurately than males and ITC individuals as heterospecific heterosexual pairs were rare phenomenon while homosexual ones were not. We suggest that these potentially unproductive copulations may indicate the basal frequency of meetings between snails of different species, and thus to roughly demarcate the effects of habitat choice (potentially unproductive heterospecific matings) and mate choice (conspecific male–female matings). The correlation between the sizes of the mates was expected, but its magnitude differed across the species. Quite probably, in some species pairs, the differences in shell size of adult snails ensure reproductive isolation based on mate choice mechanisms. Nevertheless, considering the data on microhabitat preferences^28^, we suggest that the principal proximate mechanism of PMI in the sympatric species group studied is the habitat choice (including habitat-preference and habitat associated fitness isolation). This mechanism makes the strongest contribution, but can be complemented by others. This becomes evident when all types of naturally occurring pairs (with females, males, immature and castrated con- and heterospecific individuals) are considered.

Both homo- and heterosexual copulations were registered in four of the five species. It has previously been shown that the heterosexual copulation lasts much longer than the homosexual one^38,39^. This can lead to an underestimation of the observed frequency of homosexual copulation^38^. Moreover, the duration of copulations with conspecific females depends on their size and shell shape^38,50^. On the other hand, snails engaged in shorter copulations have a chance to make more mating attempts. This means that these two factors are opposite. Thus, we assumed that the number of registered pairs in our study does reflect the frequencies of mating attempts of the males, which are the active sex in snails, while females are passive during copulation^29–31^.

### Male-male mating phenomenon

Male-male pairs have been recorded fairly often in studies of mating activity of littorinids^37,38,40,42,43,60^. In some species, *e.g. L. saxatilis*, the rate of homosexual mating reached 30%^42^, and a biological significance of this phenomenon was suggested^38^. Other littorinids, such as *Nodilittorina hawaiiensis* or *Littoraria pintado*, have never been detected in male-male pairs even when a large number of pairs were analysed^79^.

The species in our study varied significantly in their propensity to form male-male pairs (Table 2). The mean frequency of male-male copulation was about 16–18% in *L. saxatilis*, *L. compressa* and *L. obtusata.* It was much higher, up to 36%, in *L. arcana* and presumably hybrid males (with 1.5 penial rows), while *L. fabalis* males were not found in homosexual pairs at all. This distinctiveness of *L. fabalis* was quite unexpected because males of both *L. fabalis* and *L. obtusata* were shown to discriminate the sex of passive partners by their mucus trails, while males *L. saxatilis* were not (Johannesson et al., 2010). Importantly, sexes of these species did not differ in shell shape^28^.

### Premating isolation between the “saxatilis” and “obtusata” groups

Almost no potentially productive copulations were registered between members of different groups. This suggests that there is a reliable premating isolation between the “saxatilis” and the “obtusata” group of cryptic species. The rare exceptions were represented by sporadic *L. fabalis*/*L. saxatilis* pairs (in both directions). Quite possibly, the involvement of *L. saxatilis* into these pairs was associated merely with the fact that it was the most abundant “saxatilis” species. Interestingly, *L. fabalis* males were also collected in pairs with *L. obtusata* females, while *L. obtusata* males were not encountered with either *L. saxatilis* or *L. fabalis* females. In contrast, both *L. obtusata* and *L. saxatilis* males were collected in homosexual heterospecific (between-group) pairs, but the males of *L. fabalis* were not. This illustrates the differences in behavioural biology between these species (see below).

### Premating isolation within the “obtusata” group

The pattern of mate choice of *L. fabalis* in the populations studied may be considered as adaptive since most of the copulations were potentially productive. It proved to be well-isolated from *L. obtusata*, which is supported by high values of I_PC_ and both female and male FI values. An effective PMI between *L. fabalis* and *L. obtusata* observed in our study agrees well with the results of genetic analyses showing a clear differentiation between these species^80–82^, with the exception of one population in Portugal^83^. Typical proteomes and metabolomes of *L. fabalis* and *L. obtusata* also show robust differences (in contrast to *L. saxatilis vs L. arcana*^28,84^). This phenomenon might also be related to effective reproductive isolation.

The observation of *L. fabalis* males with *L. obtusata* females (but not vice versa) in all three years of the analysis corresponds to the propensity of *L. fabalis* males to follow large females^41,46^. This is in line with the general tendency in gastropods and other invertebrates: males tend to be smaller than the females they mate^85^. Accordingly, in the populations analysed, *L. obtusata* females in copulating pairs were larger than the active *L. fabalis* males, which in general mated with larger passive partners. *L. obtusata* males also mated with slightly larger partners while *L. fabalis* females were mainly a bit smaller (Fig. 4). This attests that the shell size contributes to PMI between these two species, even though to a lesser extent than in the case of *L. compressa vs L. arcana* and *L. saxatilis*. The importance of size in the mate choice was also suggested to be a background for the reduced gene flow between *L. fabalis* ecotypes in Swedish populations^46,86^. These observations support the hypothesis that a shift in mean size, due to growth rate or maturation time, may facilitate the divergence of sister species in snails as it contributes to assortative mating within diverging morphs^31,43,46^.

Although shell size obviously contributes to mate choice in *Littorina* species, it cannot play a leading role in PMI due to significant overlap in size between the species, especially considering that copulations with young individuals are common. Other mechanisms such as differences in shell shape^28,33,87^ are probably involved as well*.* Nevertheless, it seems reasonable to conclude that the major factor preventing interspecies mating is differentiation of ecological niches: in populations of the studied region *L. obtusata* prefers to keep during low tide within *A. nodosum* canopy, while *L. fabalis* is mainly associated with open parts of *F. serratus*^28^. Together with a low dispersal ability, this differentiation provides a strong precondition for “by species”-assortative mating. The acquisition of different ecological preferences was probably a crucial event in the divergence between these species.

At the same time, premating barriers between *L. fabalis* and *L. obtusata* are not absolute, and some postmating mechanisms such as sperm competition or cryptic female choice should exist. Noteworthy, a protein factor presumably involved in PMPZ barriers (LOSP) was recently described in *L. obtusata* parasperm cells^88,89^. These cells together with attached spermatozoa bunches are transferred to the female during insemination and stored within the receptacle^33,90^. Paraspermal secretory proteins affect sperm survival, motility and fertilization success. Significant differences in LOSP structure were identified between *L. fabalis* and *L. obtusata*^91^, indicating its potential role in the maintenance of the reproductive barriers between these species.

### Premating isolation within the “saxatilis” group

Within the “saxatilis” group of cryptic species, *L. compressa* snails were highly species-specific in copulations, in contrast to *L. saxatilis* and *L. arcana*. Differences in the microbiotopic distribution between *L. compressa* on the one hand and *L. arcana* and *L. saxatilis* on the other hand^28,36^ are the major factor separating *L. compressa* and uniting *L. saxatilis* and *L. arcana* in their copulative patterns.

Nonetheless, *L. compressa* populations spatially overlap with those of *L. saxatilis* and *L. arcana* along the intertidal zone. The shell size differences, again, fortify PMI. Indeed, copulating *L. compressa* individuals were significantly smaller than those of *L. saxatilis* and *L. arcana* (Fig. 4). The snails following the trail of a prospective mate^92^ can perceive chemical and mechanical cues, including the size of the snail that left the trail^45,93^. Pronounced differences in the size and the shape of the shell between the wave and the crab ecotypes of *L. saxatilis* are supposed to drive strong assortative mating^31,50,94–96^. In contrast, the ecotypes of *L. fabalis* are not so strongly differentiated by size and have a rather weak mating barrier^46^. Similarly, size differences might contribute to a mating barrier between *L. compressa* and *L. saxatilis*/*L. arcana* species pair.

### The “arcana/saxatilis” paradox

*Littorina arcana* and *L. saxatilis* are a notable case, contrasting with the initial expectation of strong PMI between sympatric sister-species. These species are ecologically very similar (at least in high-shore^28^), and there are no records of *L. arcana* populations that are not sympatric with *L. saxatilis*^33^. These species resemble each other in shell shape, physiologically and genetically, and their populations overlap significantly as well^28,34,36,59,61,84,97^. In the populations studied, copulating individuals of these species were of a similar size and the males of these two species copulated with similar sets of passive partners, suggesting that PMI is weak. Frequent interspecific *L. saxatilis*/*L. arcana* copulations are probably associated with the lack of prominent ecological and shell size differences. Importantly, mating interactions between these species were asymmetric. Females of *L. arcana* were largely ignored by the males of both *L. arcana* and *L. saxatilis* (as well as by *L. saxatilis* 1.5 row males). Out of the 25 pairs involving *L. arcana* males, only three pairs involved *L. arcana* females (Table 2). A low proportion of *L. arcana* females engaged in mating is striking, because the sex ratio in the analysed *L. arcana* populations was in favour of the females (Supplement S3,S4).

*Littorina arcana* females were the rarest passive partners in the all pairs, including *L. arcana* and/or *L. saxatilis* (Table 2) and as a consequence had a negative FI value (Supplement S7). A possible explanation of this pattern is that *L. arcana* females, though morphologically indistinguishable, were represented by a mixture of “pure” and “hybrid” (*L. arcana*/*L. saxatilis*) individuals in the populations under study. These “pure” and “hybrid” individuals significantly varied in their attractiveness for the males of both *L. arcana* and *L. saxatilis*. This interpretation is compatible with the results of Warwick et al.^63^, who showed that hybrid “arcana/saxatilis” females (resulting from breeding of *L. saxatilis* males with *L. arcana* females but not vice versa) had *L. arcana* morphology.

This hypothesis is supported by direct and indirect evidence on the possibility of asymmetric hybridization between these species. The possibility of one-directional hybridization between them (but not with *L. compressa* in any combination) was first demonstrated in laboratory experiments that yielded viable and fecund offspring with normal segregation of parental alleles in the next generations^63,98^. The distribution pattern of the *L. arcana* specific locus A2.8 (revealed by comparative RAPD analysis) also implied the possibility of limited gene flow between *L. arcana* and *L. saxatilis* and the existence of viable hybrids in the wild^61^. An exhaustive analysis of microspatial distribution of these two species and their presumably hybrids across a vertical shore gradient showed a clear correlation between the frequencies of *L. arcana* and presumably hybrids in populations. Interestingly, the interspecific gene flow was shown to fit an asymmetric model, where both “pure” *L. saxatilis* and rare hybrid “arcana/saxatilis” females were involved in hybridization, while “pure” *L. arcana* females were not^36^. Thus, the *L. arcana* females engaged in the copulations in this study might correspond to “hybrid” females while the rest of the females, ignored by males (e.g., due to reproductive seasonality), could be “pure” *L. arcana* females. This hypothesis might be verified in studies involving genome-wide analyses to qualify true differences between morphologically indistinguishable *L. arcana* and hybrid females. Our interpretations are in concordance with the predictions by Warwick et al.^63^ that the percentage of hybrid “arcana/saxatilis” females in the wild populations should be low, not more 2%. An asymmetric genome-wide introgression from *L. saxatilis* to *L. arcana* was also inferred in a recent study^64^, though the authors did not reveal any direct interspecies hybrids among 3092 genotyped individuals, which was possibly a specific feature of the populations studied.

Noteworthy, the gap in PMI in the nature observed in our study (*L. arcana* males actively mate with *L. saxatilis* females) was an exact opposite of the gap in PMPZ isolation detected in the laboratory (*L. saxatilis* successfully cross with *L. arcana* females, but not reciprocally). This resembles an asymmetric PMI between allopatric populations of *Drosophila montana*^15^, where the pre- and postmating barriers compensated for each other’s weakness: PMPZ were effective only in combinations with weak PMI and vice versa. In the case of *L. arcana*/*L. saxatilis* species pair, several reproductive barriers function asymmetrically as well. Some of them are weak, eventually allowing some leakage of genes from *L. arcana* to *L. saxatilis* (as revealed with A2.8 marker^61^). It was demonstrated that species isolated by a large number of weak reproductive barriers have a more complicated genetic architecture than strongly isolated ones^99^. This forms a favourable background for a detailed comparative analysis of *L. saxatilis* populations either accompanied or not by *L. arcana*, including aspects of potential PMPZ isolation mechanisms. A paraspermal protein LOSP, potentially involved in the maintenance of postcopulatory reproductive barriers, was recently characterized in the *Littorina* (*Neritrema*) species^88,89^. Noteworthy, this protein demonstrates a high degree of polymorphism in the *L. saxatilis*/*L. arcana* pair, where PMI is weak, unlike *L. obtusata*, which is well isolated from the other species at the premating level^91^.

## Summary, conclusions and perspectives

We analysed the premating reproductive isolation in a post-speciation model involving a group of recently diverged sympatric species. We showed that five evolutionarily young species do vary in the strength of PMI. With one notable exception, heterospecific heterosexual copulations were not typical though they do occur in the wild at a low rate. Our results are particularly interesting in the context of an upcoming study, where we demonstrate clear differences in microhabitat preferences for all species pairs except *L. arcana*/*L. saxatilis*^28^. We previously compared molecular phenotypes in the context of phylogenetic closeness of those species, with an exceptionally high degree of similarity between *L. saxatilis*/*L. arcana*^84^. Species of this pair have similar ecological preferences in the upper intertidal level, and their males copulate with similar sets of passive partners. Finally, there is a clear correspondence between the degree of differentiation of ecological niches, the degree of molecular phenotype dissimilarity, and the efficacy of PMI. Altogether, this suggests a major, and most probably evolutionarily primary role of habitat choice in the PMI maintenance between *Littorina* species. This, in turn, lends support to the hypothesis of the origin of these species by sympatric ecological speciation with a limited gene flow via niche differentiation within the intertidal zone. Life in a certain type of microhabitat combined with a low dispersal (due to low motility and absence of planktonic larvae) possibly served as a prerequisite for assortative mating, which occurs via habitat choice. This promoted accumulation of adaptive traits such as shell shape, size, proteomic and metabolomic characteristics, while mate choice joined as a secondary acting force based on matching rule, when traits listed above were important (not only conspecific females, but also conspecific males were mated more often than heterospecific ones). Finally, these factors together strengthened the divergence between incipient species, and acted as a powerful force increasing the probability of a successful speciation event^9,10,12,100^. This scenario appears quite plausible for the European North Atlantic *Neritrema* species with clearly diverged ecological preferences. Interestingly, the species of passive partner in the *Littorina* populations studied was recognized with a greater accuracy in females than in males. This indicates that mate choice based on preference/trait rule^12^ may also contribute to PMI between *Littorina* (*Neritrema*) species.

The pair *L. arcana* and *L. saxatilis* demonstrated a peculiar mating pattern, with both species showing preference for *L. saxatilis* females. There were no differences in shell size and shape between these two species^28^. They are also similar ecologically and at proteomic and metabolomic levels^28,84,97^. Thus, habitat choice and adaptation to microenvironment hardly explain the background of divergence between these species. Importantly, the males of *L. arcana* demonstrated a significantly higher rate of both homosexual and heterospecific copulations among all species studied. This implies that (1) the males of this species are more indiscriminate in their choice of passive partners, causing inefficacy in PMI and (2) other barriers, *e.g.,* some mechanisms of PMPZ such as differential sperm viability, motility, transfer, storage or usage, physical and chemical cues interfering sperm guidance, gamete fusion, and eventual formation of a zygote^101^ may play a crucial role in the divergence of this species pair. Thus, the analysis of PMPZ mechanisms in the *Littorina* (*Neritrema*) species is a promising direction of future studies.

## Supplementary Information

Supplementary Information.
