# Supplementary material for: Premating barriers in young sympatric snail species
Source: Sci Rep. 2021 Mar 11;11:5720. doi: 10.1038/s41598-021-84407-2 (PMC7952697; doi:10.1038/s41598-021-84407-2)
Supplement: Supplementary file 1 — Supplementary Information. [file 41598_2021_84407_MOESM1_ESM.pdf]

Premating barriers in young sympatric snail species

Arina L. Maltseva[1]*, Marina A. Varfolomeeva[1], Arseniy A. Lobov[1,2], Polina O. Tikanova[1,3], Egor A. Repkin[1], Irina Y. Babkina[1], Marina Panova[1,4], Natalia A. Mikhailova[1,5] and Andrei I. Granovitch[1]

1 Department of Invertebrate Zoology, St Petersburg State University, St Petersburg, Russia
2 Laboratory of Regenerative Biomedicine, Institute of Cytology Russian Academy of Sciences, St Petersburg, Russia
3 Institute of Molecular Biotechnology of the Austrian Academy of Sciences (IMBA), Vienna, Austria
4 Department of Marine Sciences - Tjärnö, University of Gothenburg, Sweden
5 Centre of Cell Technologies, Institute of Cytology Russian Academy of Sciences, St Petersburg, Russia

* Corresponding author: arina.maltseva@spbu.ru;

ABSTRACT

Sympatric coexistence of recently diverged species raises the question of barriers restricting the gene flow between them. Reproductive isolation may be implemented at several levels, and the weakening of some, *e.g.* premating, barriers may require the strengthening of the others, *e.g.* postcopulatory ones). We analysed mating patterns and shell size of mates in recently diverged closely related species of the subgenus *Littorina Neritrema* (Littorinidae, Caenogastropoda) in order to assess the role of premating reproductive barriers between them. We compared mating frequencies observed in the wild with those expected based on relative densities using partial canonical correspondence analysis. We introduced the fidelity index (FI) to estimate the relative accuracy of mating with conspecific females and precopulatory isolation index (I_{PC}) to characterize the strength of premating barriers. The species under study, with the exception of *L. arcana*, clearly demonstrated preferential mating with conspecifics. According to FI and I_{PC}, *L. fabalis* and *L. compressa* appeared reliably isolated from their closest relatives within *Neritrema*. Individuals of these two species tend to be smaller than those of the others, highlighting the importance of shell size changes in gastropod species divergence. *L. arcana* males were often found in pairs with *L. saxatilis* females, and no interspecific size differences were revealed in this sibling species pair. We discuss the lack of discriminative mate choice in the sympatric populations of *L. arcana* and *L. saxatilis,* and possible additional mechanisms restricting gene flow between them.

Keywords: copulation, cryptic species, *Littorina*, mating behaviour, premating reproductive isolation, sympatry, assortative mating

Supplement_1. Subdivision of the intertidal area into two parts (levels) in the example of the site Varanger-fjord, Kiberg. The lower part of the intertidal area (width ~ 25 m) was distinguished based on biocenotic characteristics: it was heavily overgrown by fucoid macroalgae; the species of fucoids alternated in a sequence (from the most lower level upwards) *Fucus serratus* -> *Ascophyllum nodosum* -> *Fucus vesiculosus*. The upper part of the intertidal zone was ~ 1.5 times narrower and included large boulder and rocky ledges without macrophytes with smaller boulders and pebbles partially covered with scattered clumps of *F. vesiculosus*. These two parts of the littoral zone did not contact and were separated by a belt of 3-5 m width.

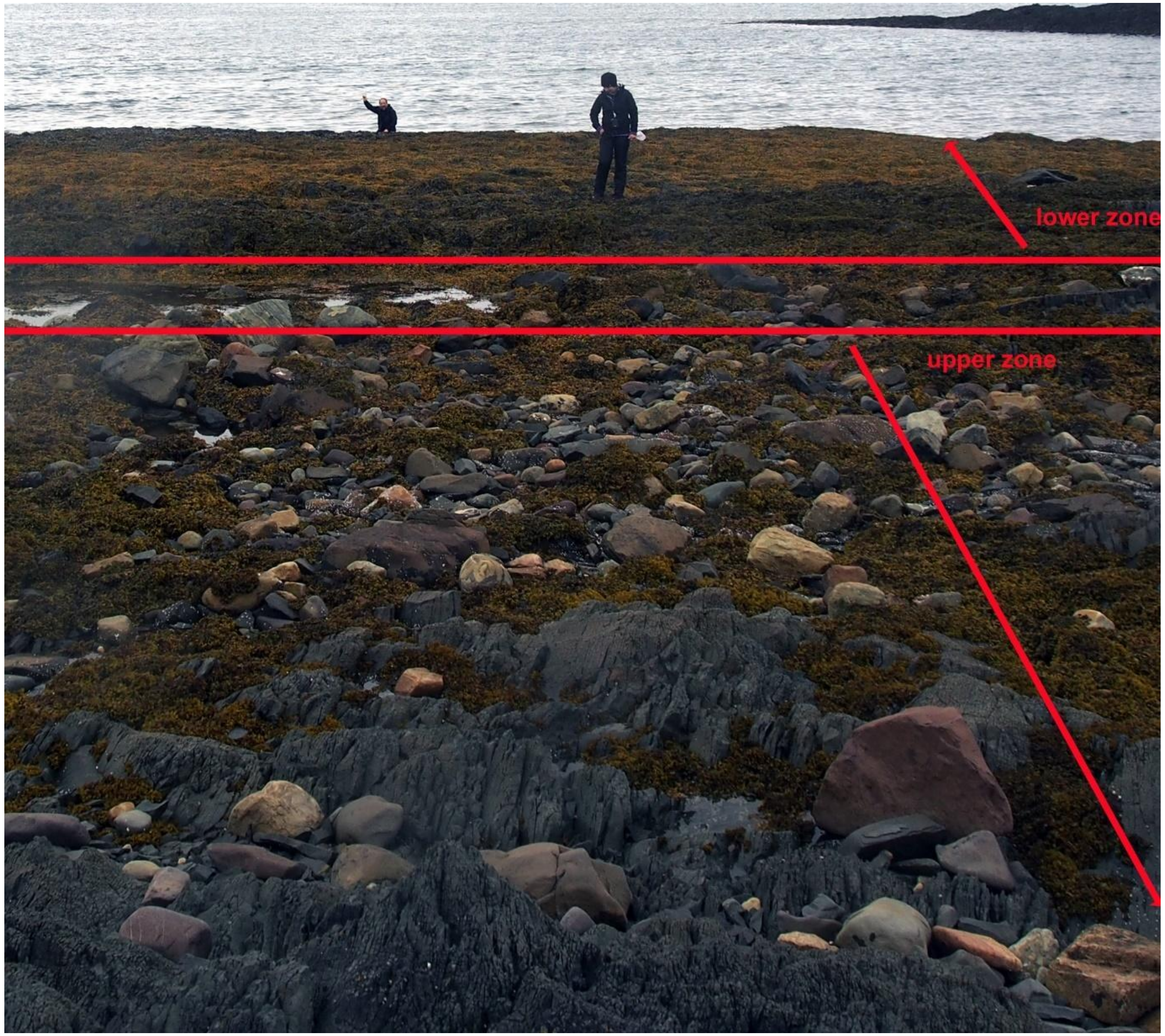

Supplement_2. Procedure of verification of a copulation attempt in the example of the *Littorina obtusata* pair. The pair was caught in a typical position and examined to verify that the penis of the active partner (the snail held by fingers in A-G) was inserted into the mantle cavity of the passive partner (the snail held by forceps in A-G). When the pairing partners were separated from each other, the copulatory organ (marked by a blue arrow) became visible while appearing from the mantle cavity of the passive partner.

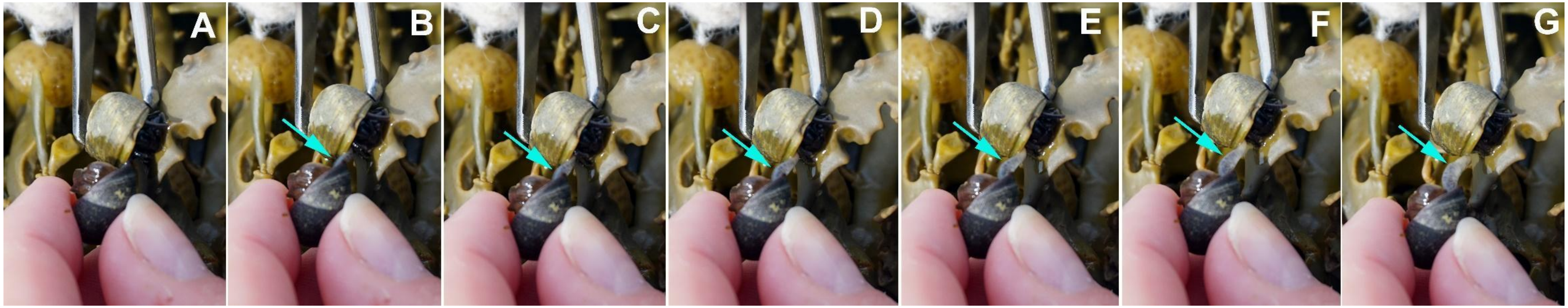

Supplement_3. Average relative densities of Littorina snails grouped by site (year) and part of the intertidal zone. Snails of different species, sex, and maturity are shown by different colours corresponding to the legend. The categories "obtusata" and "saxatilis" include immature or castrated individuals of the corresponding cryptic species group (as such individuals can be unambiguously identified to species group only). Relative densities are presented in the Supplement_4.

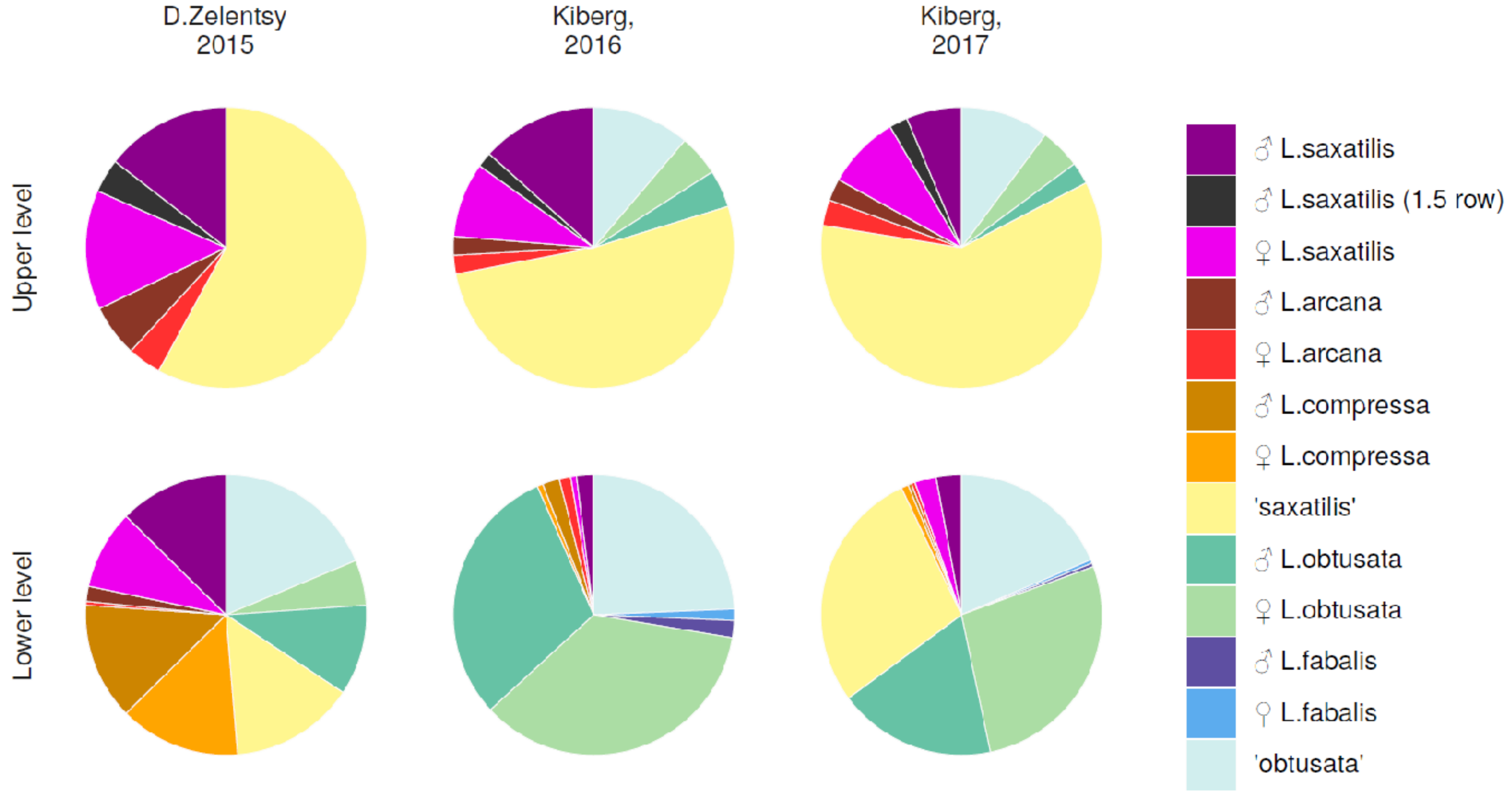

Supplement_4. Mean relative densities by site/year, intertidal level, species and sex. Site/year: DZ2015 – D.Zelentsy 2015; K2016 and K2017 – Kiberg 2016 and 2017 respectively. Species: arc – *L. arcana*, comp - *L. compressa*, sax – *L. saxatilis*, sax 1.5 – *L. saxatilis* males with 1.5 rows of mamilliform penial glands, s-like – immature or castrated individuals of the “saxatilis” cryptic species group, fab – *L. fabalis*, obt – *L. obtusata*, o-like - immature or castrated individuals of the “obtusata” cryptic species group; sex: f – female, m – male, ITC – immature of trematode castrated.

Site/year	Level	Species	Sex	Mean density, %	SD	Site/year	Level	Species	Sex	Mean density, %	SD
**DZ2015**	upper	sax	f	14.20	0.05	K2016	lower	comp	f	16.70	
**DZ2015**	upper	sax	m	13.00	0.05	**K2016**	lower	comp	m	18.30	0.02
**DZ2015**	upper	sax 1.5	m	5.00	0.01	**K2016**	lower	obt	f	36.10	0.08
**DZ2015**	upper	s-like	ITC	60.10	0.06	**K2016**	lower	obt	m	26.40	0.09
**DZ2015**	upper	arc	f	4.70	0.01	**K2016**	lower	fab	f	11.30	0.12
**DZ2015**	upper	arc	m	5.90	0.04	**K2016**	lower	fab	m	10.30	0.09
**DZ2015**	lower	sax	f	10.20	0.06	**K2016**	lower	o-like	ITC	22.80	0.14
**DZ2015**	lower	sax	m	11.50	0.08	**K2017**	upper	sax	f	8.80	0.04
**DZ2015**	lower	s-like	ITC	17.90	0.10	**K2017**	upper	sax	m	8.80	0.02
**DZ2015**	lower	arc	f	2.30		**K2017**	upper	sax 1.5	m	4.60	0.02
**DZ2015**	lower	arc	m	2.40	0.01	**K2017**	upper	s-like	ITC	61.00	0.12
**DZ2015**	lower	comp	f	14.90	0.10	**K2017**	upper	arc	f	8.90	0.05
**DZ2015**	lower	comp	m	14.10	0.09	**K2017**	upper	arc	m	4.20	0.01
**DZ2015**	lower	obt	f	7.20	0.02	**K2017**	upper	obt	f	10.10	0.04
**DZ2015**	lower	obt	m	13.30	0.09	**K2017**	upper	obt	m	4.90	0.02
**DZ2015**	lower	o-like	ITC	17.80	0.05	**K2017**	upper	o-like	ITC	13.20	0.09
**K2016**	upper	sax	f	9.50	0.03	**K2017**	lower	sax	f	5.70	0.03
**K2016**	upper	sax	m	14.00	0.09	**K2017**	lower	sax	m	7.30	0.01
**K2016**	upper	sax 1.5	m	4.80	0.02	**K2017**	lower	s-like	ITC	44.70	0.25
**K2016**	upper	s-like	ITC	49.40	0.13	**K2017**	lower	arc	f	3.80	
**K2016**	upper	arc	f	3.20	0.01	**K2017**	lower	comp	f	3.30	0.00
**K2016**	upper	arc	m	3.00	0.01	**K2017**	lower	comp	m	2.90	
**K2016**	upper	obt	f	7.00	0.03	**K2017**	lower	obt		50.00	
**K2016**	upper	obt	m	8.90	0.02	**K2017**	lower	obt	f	33.60	0.21

**K2016**	upper	o-like	ITC	13.20	0.05	**K2017**	lower	obt	m	22.20	0.15
**K2016**	lower	sax	f	10.00		**K2017**	lower	fab	f	10.00	
**K2016**	lower	sax	m	16.10	0.11	**K2017**	lower	fab	m	3.30	
**K2016**	lower	arc	f	7.00	0.04	**K2017**	lower	o-like	ITC	24.20	0.14

Supplement_5. The number of copulations in which Littorina snails of a given species were involved. All the categories of pairs were counted (con- and heterospecific, homo- and heterosexual). $\mathbf{N_{total}}$ - total number of pairs includes all pairs where any of the partners belonged to a given species. $\mathbf{N_{active}}$, $\mathbf{N_{passive}}$ - number of pairs as active (or passive) partner includes all pairs (homo- and heterosexual) in which active (or passive) partner belonged to a given species. The category *L. saxatilis* (1.5 row) includes male *L. saxatilis* with 1.5 rows of penial glands (presumable hybrids of *L. saxatilis* and *L. arcana*). The categories “obtusata” and “saxatilis” include immature or castrated individuals of the corresponding cryptic species group.

	Number of pairs		
**Species**	N_{total}	N_{active}	$N_{passive}$
L. saxatilis	181	142	138
***L. saxatilis* (1.5 row)**	32	27	7
L. arcana	34	29	8
L. compressa	11	9	11
L. obtusata	91	86	87
L. fabalis	25	24	18
**'saxatilis'**	42	-	42
**'obtusata'**	6	-	6

Supplement_6. Matings of females by site and species. The mating patterns of the female *Littorina* snails grouped by site, intertidal level and species. Total number of copulations of a particular female species, as well as the number (and percentage) of copulations grouped by active partner category is given. Both *L. saxatilis* and *L. arcana* females *were* considered as conspecifics of *L. saxatilis* males with 1.5 rows of mamilliform penial glands. Empty table cells indicate that corresponding mating combinations were not detected.

Site / Year	Level	Female species	Total	Same-species male	Other-species male
D. Zelentsy, 2015	upper	*L. saxatilis*	54	41 (75.9)	13 (24.1)
	lower	*L. saxatilis*	13	10 (76.9)	3 (23.1)
		L. compressa	4	3 (75)	1 (25)
		L. obtusata	19	19 (100)	
		L. fabalis	1	1 (100)	
Kiberg, 2016	upper	*L. saxatilis*	15	13 (86.7)	2 (13.3)
	lower	*L. saxatilis*	15	9 (60)	6 (40)
		L. compressa	1	1 (100)	
		L. obtusata	28	27 (96.4)	1 (3.6)
		L. fabalis	2	2 (100)	
Kiberg, 2017	upper	*L. saxatilis*	10	8 (80)	2 (20)
		L. arcana	3	1 (33.3)	2 (66.7)
		L. obtusata	7	7 (100)	
	lower	*L. saxatilis*	2	1 (50)	1 (50)
		L. compressa	4	4 (100)	
		L. obtusata	17	17 (100)	
		L. fabalis	15	14 (93.3)	1 (6.7)

Supplement_7. Fidelity index (FI) of female *Littorina* snails grouped by species, intertidal level and year/site. FI measures the degree of prevalence of mating of females with conspecific males. It is computed as difference of observed and expected frequencies of heterosexual conspecific copulations with females of a given species, divided by the total number of copulations with females of that species. Values of FI vary from -1 – avoidance, through 0 – random mating, to 1 – assortative mating. Pairs with *L. saxatilis* 1.5 row males were excluded from the analysis since it is uncertain which females are conspecific to them. Dashes indicate the cases, where it was impossible to calculate FI.

Species	Level	D. Zelentsy, 2015	Kiberg, 2016	Kiberg, 2017
L. saxatilis	upper	0.57 ± 0.06, p < 0.001	0.71 ± 0.1, p < 0.001	0.58 ± 0.16, p = 0.005
	lower	0.68 ± 0.12, p < 0.001	0.53 ± 0.14, p < 0.001	0.4 ± 0.38, p = 0.619
L. arcana	upper	-	-	0.29 ± 0.3, p = 0.720
L. compressa	lower	0.58 ± 0.27, p = 0.075	0.64 ± 0.39, p = 0.404	0.92 ± 0.14, p = 0.012
L. obtusata	upper	-	-	0.85 ± 0.1, p < 0.001
	lower	0.89 ± 0.03, p < 0.001	0.55 ± 0.08, p < 0.001	0.49 ± 0.12, p = 0.005
L. fabalis	lower	-	0.76 ± 0.29, p = 0.134	0.91 ± 0.07, p < 0.001

Supplement_8. Partial Canonical Correspondence Analysis of Littorina mating patterns. The biplots illustrate the effect of the type of the passive partner (its species and sex, blue regular letters) on the ordination of active partners (red bold letters) after the variation explained by the site and year was removed. A - the upper littoral zone, 149 pairs analysed; B – the lower littoral zone, 157 pairs analysed. Red bold letters indicate active partners (males of A – *L. arcana*, C – *L. compressa*, F – *L. fabalis*, O – *L. obtusata*, S – *L. saxatilis*, S1.5 – *L. saxatilis* males with 1.5 penial rows), regular blue letters – passive partners, only group centroids are shown to avoid clutter (the capital letter encodes species as above, "obt"- "obtusata"-species group, "sax" - "saxatilis"-species group; the lowercase letter encodes sex of a passive partner: m - male, f - female, i - immature or trematode-castrated. On B, males of *L. fabalis* and females of *L. arcana* were excluded from the analysis as passive partners). Remoteness from zero reflects non-randomness of mating.

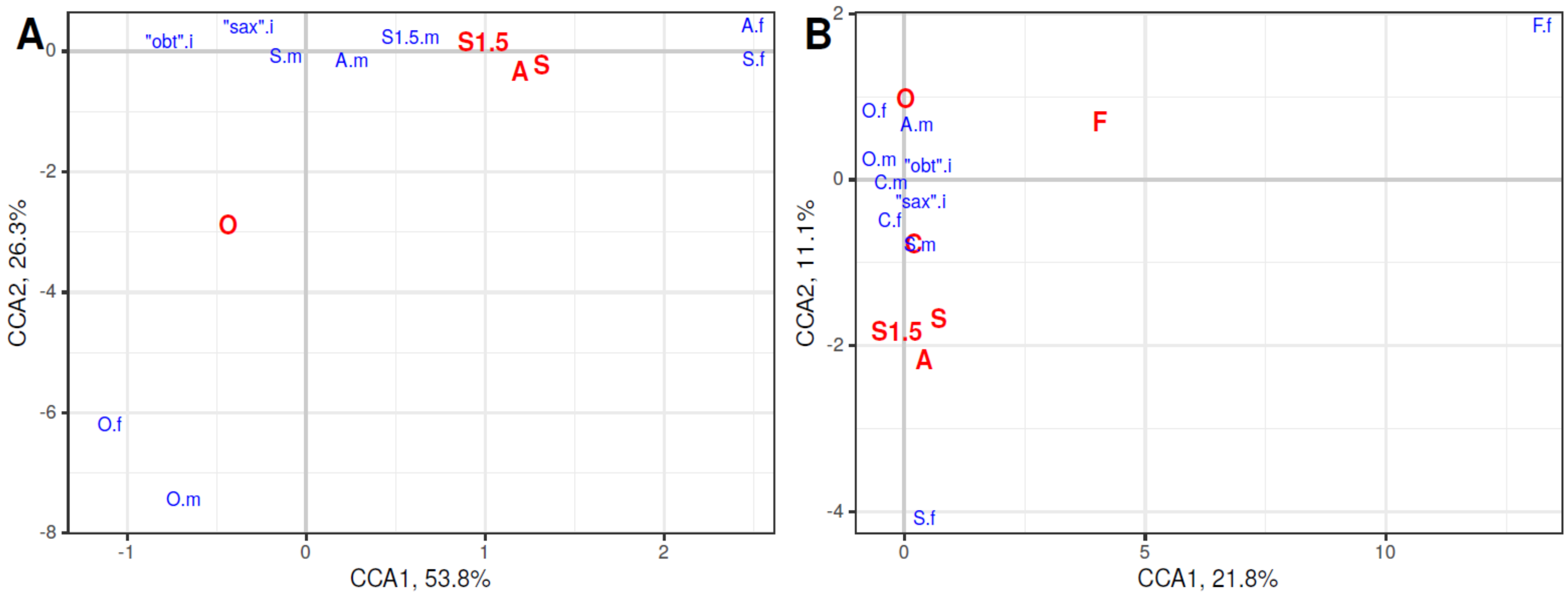

Supplement_9. Shell height [mm] of mating and non-mating Littorina snails categorized by species, year/site, and intertidal level (mean ± standard deviation). Male-male pairs are excluded.

Level	Species	Sex	D. Zelentsy, 2015				Kiberg, 2016				Kiberg, 2017			
			non-mating		mating		non-mating		mating		non-mating		mating	
			N	Size	N	Size	N	Size	N	Size	N	Size	N	Size
upper	*L. saxatilis*	female	39	8.8 ± 1.6	54	10.1 ± 1.7	20	7.6 ± 1.7	15	7.9 ± 1.3	22	7.5 ± 2.1	10	8.6 ± 4.1
	L. saxatilis	male	41	8.2 ± 1.3	51	9.8 ± 1.2	31	7.7 ± 1.8	19	7.3 ± 2	17	7.9 ± 1.6	18	7.9 ± 1.5
	L. saxatilis 1,5	male	11	9.3 ± 1.6	7	9.5 ± 1.1	4	8.1 ± 2	3	9.7 ± 1.5	5	11 ± 0.9	3	9.6 ± 1.9
	'saxatilis'	ITC	164	6.6 ± 1.4	13	9.4 ± 2	120	5.8 ± 1.6	7	6.9 ± 1.2	145	6.1 ± 2	10	8.4 ± 2
	L. arcana	female	11	10.7 ± 1.8			5	9.1 ± 1.6			7	11.5 ± 0.6	3	12.2 ± 2.1
	L. arcana	male	17	9 ± 1.5	9	10.5 ± 1.1	5	9.4 ± 3			6	9.2 ± 2.1	2	8.6
	L. obtusata	female					11	8.9 ± 2.6			11	10.4 ± 2.1	7	13.3 ± 2.1
	L. obtusata	male					10	8.5 ± 1.8			6	11.3 ± 2.4	8	12.3 ± 0.7
	'obtusata'	ITC					26	6.3 ± 2.5			24	5.4 ± 2.5	1	14
lower	*L. saxatilis*	female	21	11.9 ± 2	13	12.2 ± 2.6	1	6	15	12.5 ± 2.4	6	7.1 ± 1.4	2	12.6
	L. saxatilis	male	29	9.4 ± 2.4	15	11.6 ± 1.6	3	7.6 ± 0.4	14	11.5 ± 2.2	7	8 ± 1.7	2	13.4
	L. saxatilis 1,5	male							6	13.2 ± 2.7				
	'saxatilis'	ITC	33	6.7 ± 2.7	4	12.1 ± 1.9			8	11.1 ± 2.7	68	6 ± 1.6		
	L. arcana	female	1	8.9			2	5.7			1	11.9		
	L. arcana	male	4	10.8 ± 3.3	2	9.3			3	11 ± 0.7				
	L. compressa	female	32	6.1 ± 1.6	4	8.8 ± 0.8	1	7	1	6	2	9.5	4	6.8 ± 2.2
	L. compressa	male	31	5.9 ± 1.1	3	7.6 ± 1	3	6.2 ± 1.3	1	5	1	6	4	5.7 ± 0.6

L. obtusata	female	12	11.9 ± 3.1	19	13.8 ± 1.3	54	13.4 ± 2.4	28	13.4 ± 1.7	67	11.6 ± 1.7	17	13.2 ± 1
L. obtusata	male	24	10.5 ± 3.7	19	13.7 ± 1.2	46	11.6 ± 2.9	27	13.3 ± 1.7	44	11.3 ± 2.2	18	12.8 ± 0.9
L. obtusata										8	12.3 ± 1.1		
L. fabalis	female			1	10.3	2	7.8	2	14.2	1	11.3	15	12.7 ± 1.3
L. fabalis	male			2	11.2	3	10.5 ± 3.8	4	12.5 ± 3.1	1	12	18	11 ± 1.7
'obtusata'	ITC	43	5.5 ± 2.7			37	7.6 ± 3.4	1	8.5	46	5.7 ± 1.8	4	11.9 ± 1.4

Supplement_10. Shell height of the copulating *Littorina* snails: all the partners grouped by site and level; means are given with 95% confidence intervals via non-parametric bootstrap. Male-male pairs were excluded from the analysis.

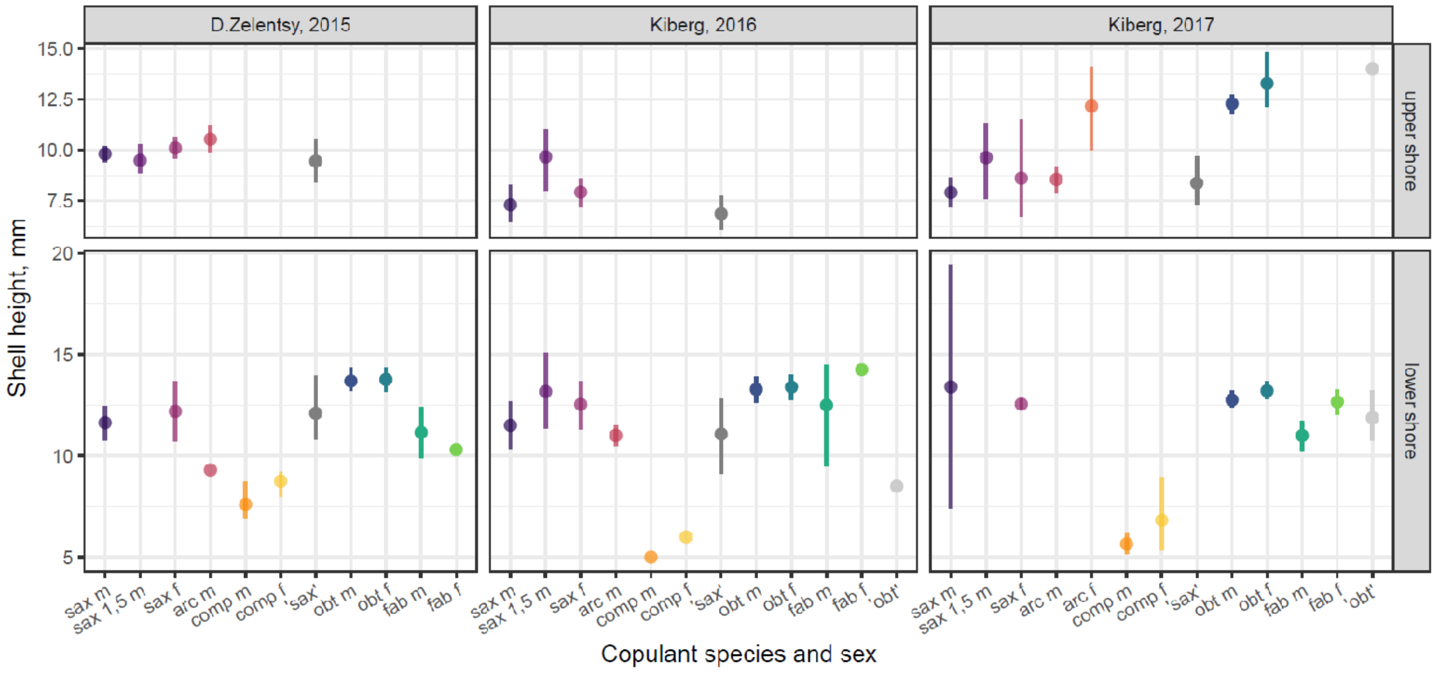

Supplement_11. Shell height [mm] of "active" males by species, pair type, year/site, and intertidal level. Means are given with 95% confidence intervals via non-parametric bootstrap. Male-male pairs are excluded. Copulants of the *L. arcana* / *L. saxatilis* species pair were smaller in the upper shore (Supplements_4,5). Therefore, tidal level should not be ignored when assessing size-assortative mating. Such tendency could not be traced for copulants of *L. obtusata*.

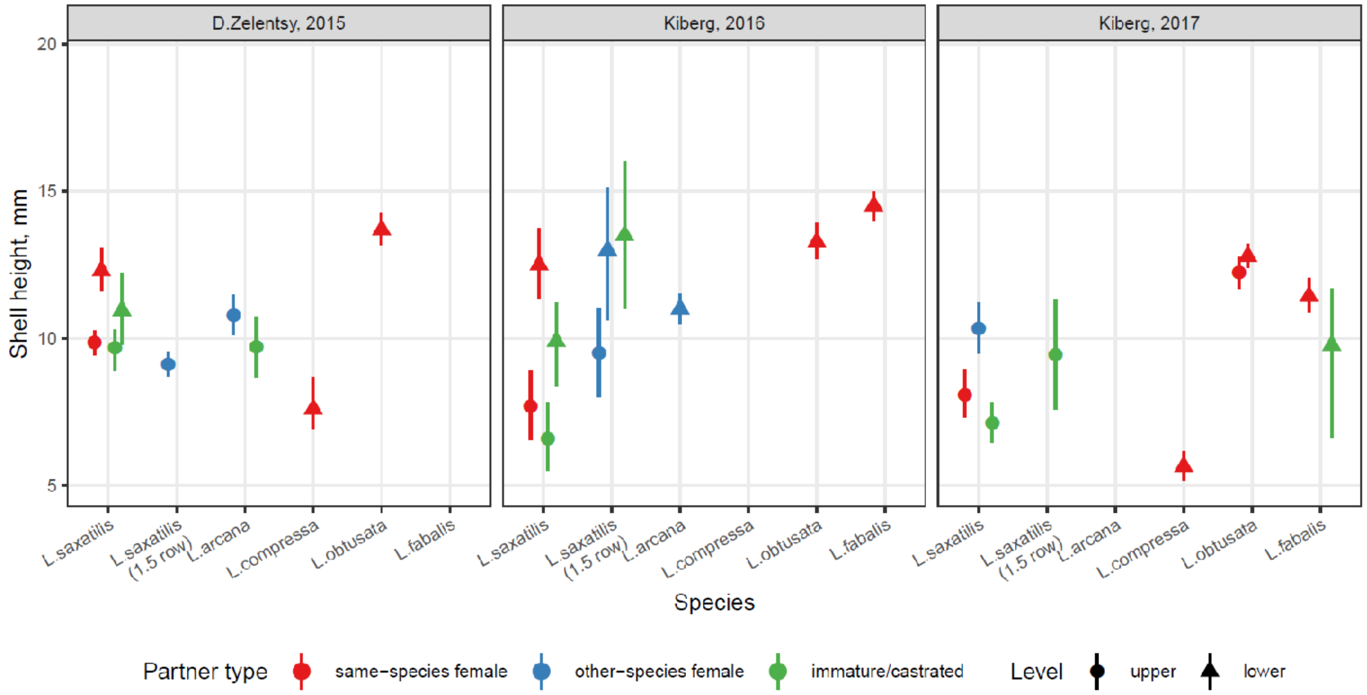

Supplement_12. Interdependence of active and passive partner sizes. Results of three-way permutational ANCOVA testing dependence of active partner size on passive partner size, pair type (same-species *vs* other), year/site and their interactions. Type III tests were run with 100 000 permutations.

Term	SS	df	F	P
Passive partner size	29	1	16	p < 0.001
Pair type	5.74	1	3.17	0.080
Passive partner size : pair type	4.4	1	2.43	0.094
Site/year	3.98	2	1.1	0.405
Passive partner size : site/year	2.6	2	0.718	0.477
Pair type : site/year	1.21	2	0.334	0.695
Passive partner size : pair type : site/year	1.19	2	0.329	0.707
Residuals	110	61		

Supplement_13. *Littorina* shell size distribution in population grouped by species and year/site. Snail copulation status (not copulating snails, copulating active males, copulating females, and males copulating with males) is shown with colour. A – upper shore level, B – lower shore level. Violins represent the density of data estimated by the kernel method. Width of the violin at any given level is proportional to relative abundance of snails with corresponding shell height. Dots are means and whiskers represent 95% confidence intervals, obtained via nonparametric bootstrap.

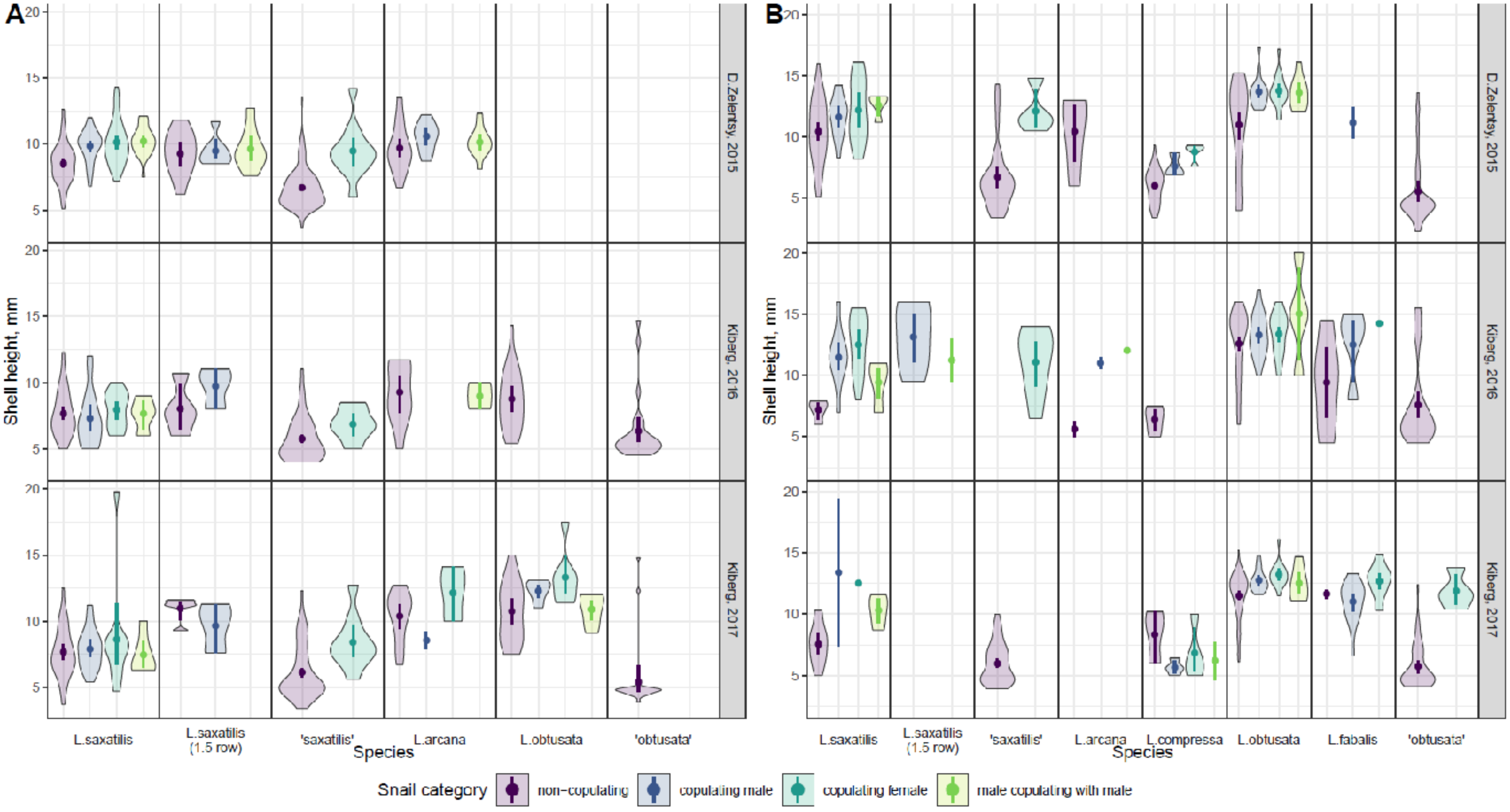

The graphs illustrate the interrelations between sizes of copulating snails and the size distribution in populations. The size of mature snails varied more broadly in non-mating individuals (sampled in square plots) than in copulating pairs. Moreover, usually snails of a bigger size were involved in copulations (the only case where this was not obvious was *L. compressa* in Kiberg 2017).

Supplement_14. Difference of partner sizes in pairs of Littorina snails ('active' - 'passive' partner size, mm). The data from different sites were pooled; the snails from the two intertidal levels were analysed separately; male-male pairs were excluded from the analysis. Numbers near the markers indicate sample size, stars indicate paired *t*-test significance (** - $p < 0.01$; * - $0.01 \leq p < 0.05$).

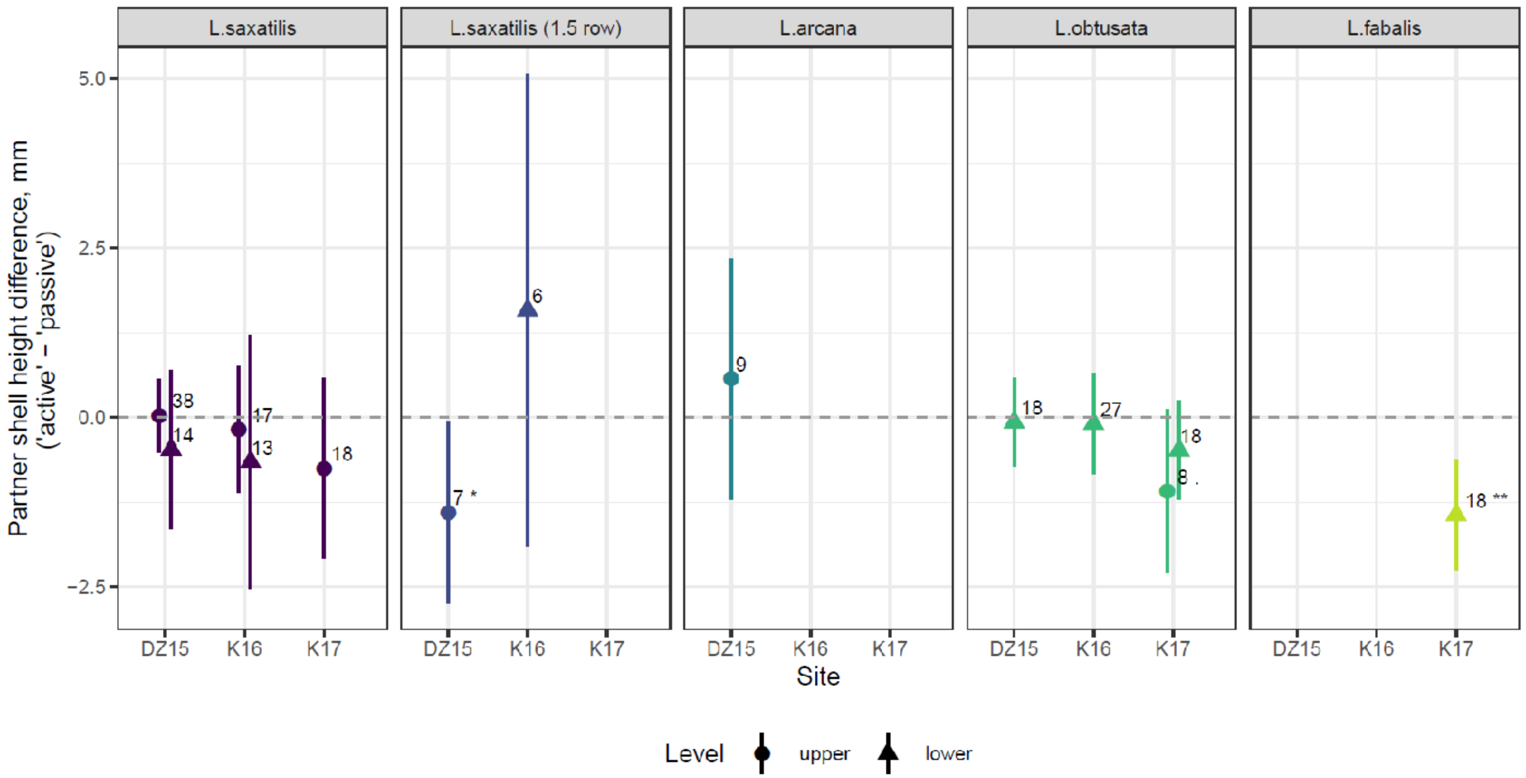

Supplement_15. Correlation of partner sizes in pairs of *L. fabalis* (navy squares), *L. obtusata* (sea green triangles) and *L. saxatilis* (larger green circles) with mature conspecific females. Regression lines are added to facilitate interpretation. In the upper level, thin green regression lines depict relationships of partner sizes in pairs of *L. saxatilis* males, when not only mature but also immature/castrated passive partners are taken into account (smaller green markers). Shaded grey areas represent 95% confidence regions for regression lines. Dashed grey line on each subplot indicates equal partner sizes: points above the line are pairs where male was larger than female, points below – *vice versa*.

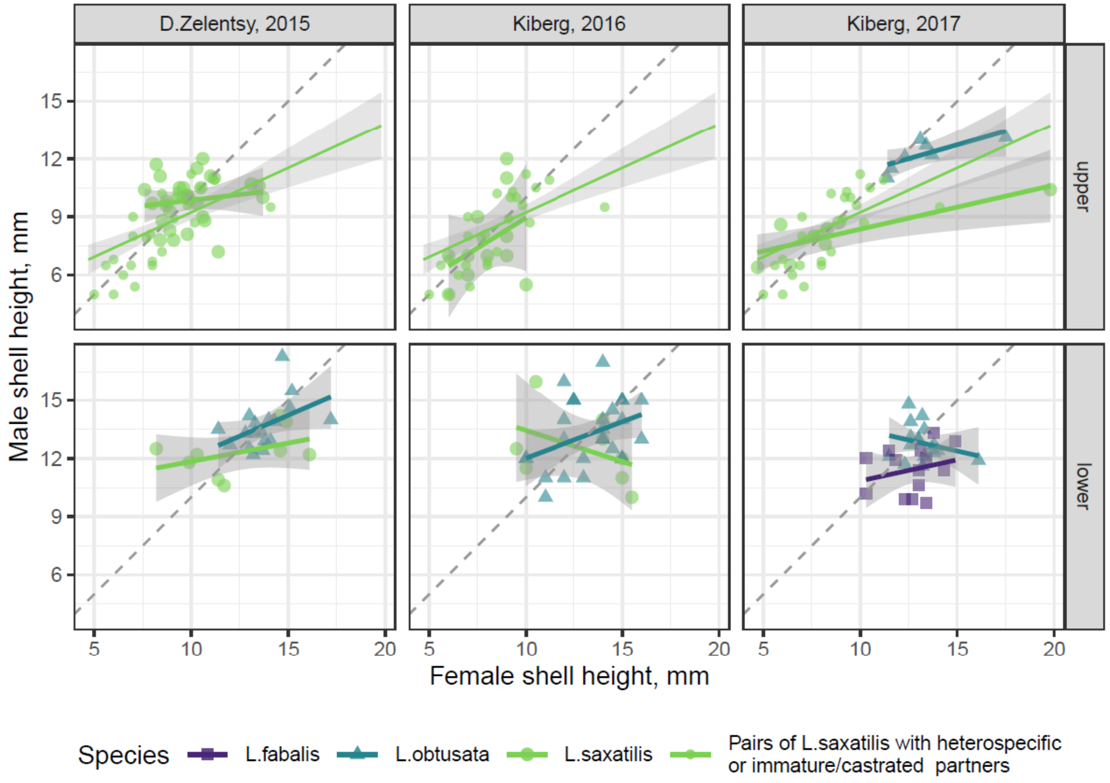

Supplement_16. Sexual isolation component for size in different populations of *L. saxatilis. L. obtusata, and L. fabalis*. N - the number of pairs with mature conspecific females in the analysis; r – Pearson correlation coefficient of partner sizes in pairs with mature conspecific females; $r > 0$ – size-assortative mating; $r = 0$ – no size assortativity; $r < 0$ – size disassortative mating (big males mate with small females and *vice versa*). p_{perm} --- two tailed p-values of rejecting the null hypothesis of absence of correlation (H_0: $r = 0$) in a permutational test with 10 000 permutations; p_{random} --- probability to get the absolute value of correlation at least this big when snails mate at random within their respective microareas (sample quadrats), that was computed from a Monte Carlo simulation with 10000 iterations; dashes indicate the cases where it was impossible to perform Monte Carlo simulation to estimate p_{random} due to low abundance of snails in the field.

Species	Site, year	Level	N	r	p_{perm}	p_{random}
L. saxatilis	D. Zelentsy, 2015	upper	29	0.15	0.43	0.78
		lower	9	0.43	0.26	0.46
	Kiberg, 2016	upper	11	0.38	0.26	0.58
		lower	8	-0.43	0.30	-
	Kiberg, 2017	upper	8	0.82	0.01	0.11
L. obtusata	D. Zelentsy, 2015	lower	18	0.46	0.07	0.37
	Kiberg, 2016	lower	27	0.38	0.05	0.29
	Kiberg, 2017	upper	7	0.76	0.02	0.90
		lower	17	-0.25	0.33	0.52
L. fabalis	Kiberg, 2017	lower	14	0.25	0.39	-
